# Identifying content-invariant neural signatures of perceptual vividness

**DOI:** 10.1093/pnasnexus/pgae061

**Published:** 2024-02-14

**Authors:** Benjy Barnett, Lau M Andersen, Stephen M Fleming, Nadine Dijkstra

**Affiliations:** Wellcome Centre for Human Neuroimaging, University College London, London WC1N 3AR, UK; Department of Experimental Psychology, University College London, London WC1H 0AP, UK; Aarhus Institute of Advanced Studies, 8000 Aarhus C, Denmark; Center of Functionally Integrative Neuroscience, 8000 Aarhus C, Denmark; Department for Linguistics, Cognitive Science and Semiotics, Aarhus University, 8000 Aarhus C, Denmark; Wellcome Centre for Human Neuroimaging, University College London, London WC1N 3AR, UK; Department of Experimental Psychology, University College London, London WC1H 0AP, UK; Max Planck UCL Centre for Computational Psychiatry and Ageing Research, University College London, London WC1B 5EH, UK; Wellcome Centre for Human Neuroimaging, University College London, London WC1N 3AR, UK

**Keywords:** awareness, perception, MEG, fMRI

## Abstract

Some conscious experiences are more vivid than others. Although perceptual vividness is a key component of human consciousness, how variation in this magnitude property is registered by the human brain is unknown. A striking feature of neural codes for magnitude in other psychological domains, such as number or reward, is that the magnitude property is represented independently of its sensory features. To test whether perceptual vividness also covaries with neural codes that are invariant to sensory content, we reanalyzed existing magnetoencephalography and functional MRI data from two distinct studies which quantified perceptual vividness via subjective ratings of awareness and visibility. Using representational similarity and decoding analyses, we find evidence for content-invariant neural signatures of perceptual vividness distributed across visual, parietal, and frontal cortices. Our findings indicate that the neural correlates of subjective vividness may share similar properties to magnitude codes in other cognitive domains.

**Significance Statement**

The vividness of conscious experience varies across different stimuli and contexts. Despite being a fundamental feature of conscious awareness, how perceptual vividness is encoded in the human brain remains unclear. Neural codes underpinning magnitude in other domains, such as reward and numerosity, have been shown to be unchanging as stimulus identity varies. In this study, we test whether components of neural activity covarying with the magnitude of perceptual vividness are similarly independent of perceptual content in analyses of magnetoencephalography and functional MRI data. We find dynamic, content-invariant neural signatures of vividness in visual, parietal, and frontal cortices. Our findings introduce the surprising notion that neural signatures of conscious experience might follow similar coding principles to magnitude properties of entirely different cognitive domains.

## Introduction

Some experiences are more vivid than others. For example, seeing a bird on a clear day will be more vivid than seeing one on a foggy evening. Similarly, a car alarm outside your office can be very vivid until your attention is consumed by a task at work. The neural correlates of experience are therefore likely to involve some representation of the magnitude of perceptual vividness. While the neural basis of perceptual vividness is yet to be systematically characterized, neural codes for magnitude quantities in other cognitive domains, such as reward and numerosity, are better understood. Many neural magnitude codes exhibit a content-invariant component, where the magnitude property is represented independently of its sensory features (1–3). For instance, the number “9” is represented as larger than the number “5,” regardless of whether we are comparing 9 vs. 5 apples, oranges, or saxophones. In this study, we ask whether the magnitude properties of perceptual vividness are also invariant to stimulus content: i.e. is the difference in vividness between seeing a bird on a clear day and a foggy evening represented in a similar manner as the difference in vividness between hearing a car alarm when we are attending to it and when we are distracted? We investigate this question by testing the extent to which neural signatures associated with reports of perceptual vividness are independent of perceptual content.

Content-invariance is a well-established feature of several neural magnitude codes. In the orbitofrontal cortex, for example, common representations of reward magnitude are shared across vastly different reward identities (1, 2, 4–6). Furthermore, presentation of the same numerosity elicits suppression effects across symbolic (Arabic numerals) and nonsymbolic (dots) stimuli in the intraparietal lobe (3), and multivariate cross-classification has revealed common representations of numerosity across symbolic and nonsymbolic formats (7). There is also evidence that numerical and reward magnitudes (among others) are encoded in a domain-general manner, where, for example, higher numbers are represented similarly to highly rewarding stimuli and lower numbers are represented similarly to stimuli with low reward values, indicating a shared neural system underpinning representations of magnitude in both domains (8–10).

**Fig. 1.**
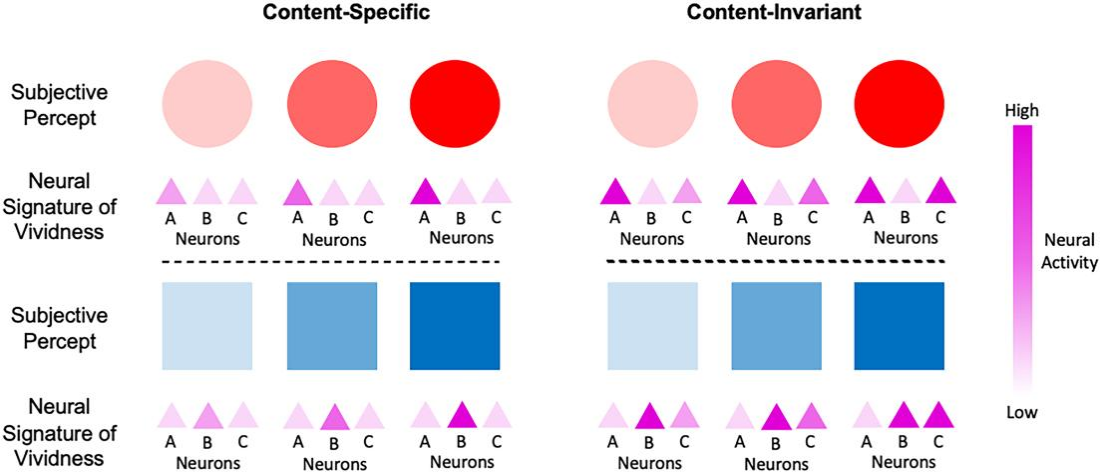
Hypothesized neural signatures of perceptual vividness. Left: Content-specific neural signatures associated with perceptual vividness. The subjective vividness of a red circle is associated with the strength of red circle–representing neurons (neuron A), while the vividness of a blue square is associated with the strength of blue square–representing neurons (neuron B). For example, as red circle–representing neurons increase their activity (top-left), the subjective percept of a red circle becomes more vivid. The neural signatures correlating with the vividness of red circles and blue squares are therefore different. Right: Content-invariant neural signatures associated with perceptual vividness. The subjective vividness of both red circles and blue squares is associated with a common neural signature (i.e. the activity of neuron C), which tracks vividness over and above any stimulus-specific neural activity (i.e. neurons A and B). Attention, emotion, and other cognitive factors may drive a content-invariant neural signal of vividness. We note that the hypothetical coding schemes represented here are not mutually exclusive, and it is possible that a combination of both schemes underpin the vividness of perceptual experience.

Given the evidence for content-invariant neural magnitude codes in other domains, it is intriguing to ask whether invariance to perceptual content is also a feature of the neural activity covarying with the magnitude of perceptual vividness. If perceptual vividness is only encoded in a content-specific manner, our experience of a stimulus, such as a red circle, may become vivid through the increased firing of neural populations representing this feature (11) (Fig. 1, left). However, if neural activity covarying with perceptual vividness also contains a content-invariant component, we should be able to find neural signatures of vividness that are independent of those covarying with sensory features. The drivers of such content-invariant signals may include changes in attention, emotion, and other cognitive factors that surpass stimulus-specific salience, but nevertheless contribute to the vividness of experience (12, 13)—an idea consistent with philosophical positions that distinguish the content of percepts from their “force” and “vivacity” (14, 15). As such, rather than being solely bound to content-specific representations, perceptual vividness might also covary with neural activity in a domain-general fashion, independently of stimulus content (Fig. 1, right) (16–19).

We note that content-specific and content-invariant coding schemes should not be viewed as mutually exclusive. For instance, it is well-known that stimulus-driven aspects of perceptual salience, such as stimulus contrast, are reflected in modality-specific neural activity (20, 21), and such properties in turn influence the subjective experience of vividness. Other studies have shown that activity in content-specific brain areas is associated with changes in perceptual awareness, even when holding the stimulus constant (22–24). Moreover, representations of magnitude in other domains, such as reward or number, often exhibit both content-specific and content-invariant components (2, 4, 7). The present study aimed to investigate whether, beyond these content-specific codes, there is also evidence for content-invariant neural signatures of perceptual vividness.

To test whether the neural code for perceptual vividness exhibits a content-invariant component, we reanalyzed both magnetoencephalography (MEG; 25) and functional MRI (fMRI; 26) data to investigate how perceptual vividness is represented in the human brain. The difficulties in isolating pure correlates of vividness and awareness from co-varying neural signals (e.g. those related to arousal or performance) are well-known, and we did not attempt to tackle these issues here (27, 28). Instead, we sought to determine the representational structure of awareness and visibility reports about different stimulus contents, to ask whether neural signatures covarying with vividness did so in a content-specific or content-invariant manner. To anticipate our results, we found evidence that neural representations of perceptual vividness generalize over stimulus content, exhibit a graded structure, and can be identified across visual, parietal, and frontal brain regions, consistent with signatures of magnitude codes in other cognitive domains.

## Materials and methods

### MEG experiment

To explore the structure and dynamics of abstract representations of awareness ratings, we reanalyzed an MEG dataset previously acquired at Aarhus University (25). The data were recorded in a magnetically shielded room using an Elekta Neuromag Triux system with 102 magnetometers and 204 orthogonal planar gradiometers. The data were recorded at a frequency of 1,000 Hz.

#### Participants

Written confirmation from the local ethics committee, De Videnskabsetiske Komitéer for Region Midtjylland, stated that the study was not subject to ethical approval under Danish law, with specific reference to Komitéloven §7 and §8.1. Nineteen participants took part in the experiment (mean age = 26.6 years; SD = 4.4 years). Two participants were excluded from our analyses: one for failing to complete the experiment and the other for not using the “almost clear experience” (ACE) rating at all (see Experimental Design and Statistical Analyses below).

#### Experimental design and statistical analyses

**Fig. 2.**
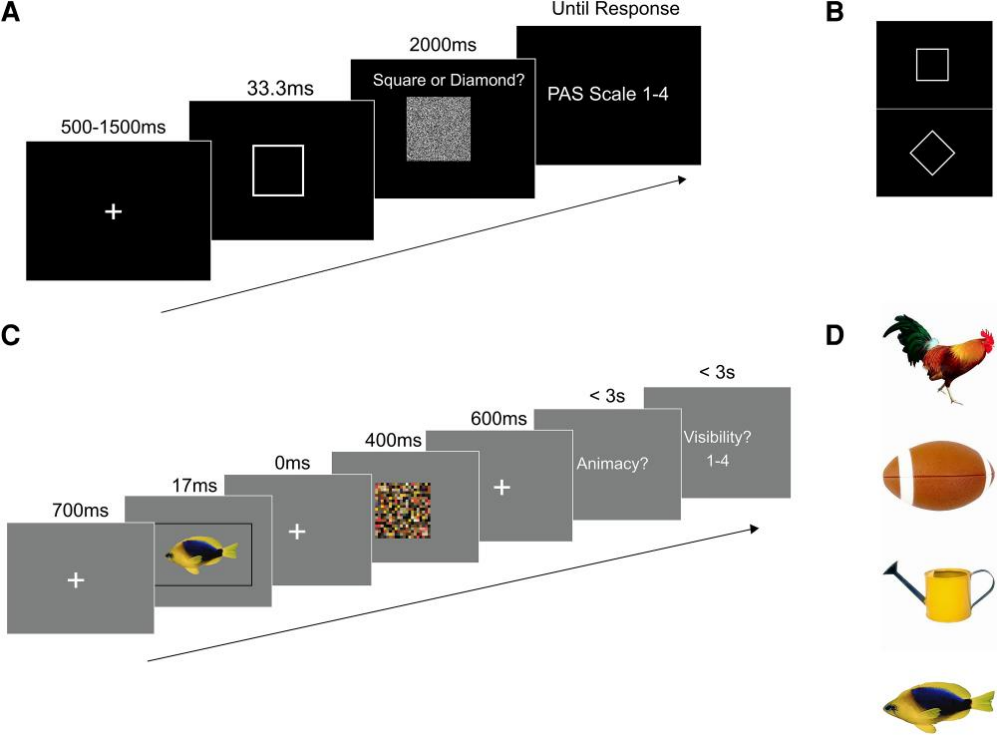
Experimental paradigms. A) Experimental paradigm for the MEG data collected by Andersen et al. (25). First, a fixation cross was presented for 500, 1,000, or 1,500 ms. Then, either a square or a diamond was shown for 33.3 ms, followed by a static noise mask for 2,000 ms. While the mask was shown, participants reported the identity of the target. Finally, they reported their awareness of the stimulus using the PAS scale. B) Stimuli used in Andersen et al. (25). C) Experimental paradigm for the fMRI data collected by Dijkstra et al. (26). A stimulus was presented for 17 ms, followed by a 66-ms ISI and a 400-ms mask. Participants then indicated whether the stimulus was animate or inanimate, and finally rated the visibility of the stimulus on a 4-point scale. D) Stimuli used in Dijkstra et al. (26).

In order to obtain a range of awareness ratings from each subject, a visual masking paradigm was used (Fig. 2A). First, a fixation cross was presented for either 500, 1,000, or 1,500 ms, followed by a target stimulus for 33.3 ms. The target stimulus was either a square or a diamond presented in white/gray on a black (RGB value 0, 0, 0) background (Fig. 2B). A static random noise mask followed the target and was presented for 2,000 ms. Participants were required to identify the target during these 2,000 ms, before rating their awareness of the stimulus on the perceptual awareness scale (PAS). PAS consists of four possible responses: no experience (NE), weak glimpse (WG), ACE, and clear experience (CE). Following identification of the target, participants reported their awareness of the stimulus. The response boxes used for target identification and awareness reports were swapped between hands every 36 trials to minimize lateralized motor responses contributing to MEG activity patterns. More details regarding the instructions given to participants about each PAS response can be found in Andersen et al. (25).

The experiment consisted of 1 practice block and 11 experimental blocks, each with 72 trials. A contrast staircase was used for the target stimuli in order to obtain a sufficient number of responses for each PAS rating. The staircase procedure had 26 contrast levels ranging from a contrast of 2 to 77%, with a step size of 3% points. In the practice block and first experimental block, the staircase increased by one level if a participant made an incorrect judgment on the identification task, and decreased by two levels if a participant made two successive correct identification judgments. For the remainder of the blocks, the staircase was adjusted based on which PAS rating the participant had used least throughout the experiment so far. Specifically, if NE had been used the least number of times throughout a block, three levels were subtracted after two consecutive correct answers, and one added for a wrong answer. If WG was the least used response, two levels were subtracted and one added. For ACE, one level was subtracted and two added. For CE, one level was subtracted and three added. This staircase procedure ensured a sufficient number of responses for each rating.

#### Preprocessing

MEG data were analyzed using MATLAB 2019a and FieldTrip (29). The data were preprocessed with a low pass filter at 100 Hz, as well as a Discrete Fourier Transform and bandstop filters at 50 Hz and its harmonics. The data were split into epochs of −200 to 2,000 ms around stimulus onset and down-sampled to 250 Hz. For baseline correction, for each trial, activity 200 ms prior to stimulus presentation was averaged per channel and subtracted from the entire epoch. During artifact rejection, trials with high variance were visually inspected and removed if they were judged to contain excessive artifacts. This procedure was performed blind to the experimental condition to avoid experimenter bias and was completed separately for the magnetometers and gradiometers in the Elekta Neuromag Triux system. Following artifact rejection, the mean numbers of trials per PAS rating were as follows (numbers in brackets refer to SDs): NE: 180.35 (59.64); WG: 168.10 (74.75); ACE: 186.35 (82.74); CE: 115.24 (77.13). To further remove eye-movement artifacts, an independent components analysis was carried out on the MEG data, and the components with the highest correlation with each of the electro-oculographic signals were discarded after visual inspection. Components showing topographic and temporal signatures typically associated with heart rate artifacts were also removed by eye.

Since we reanalyzed previously collected data, we were unable to fully control for neural signals that typically covary with awareness level. As such, to better characterize the contribution of these signals to ratings of awareness, we created two additional analysis pipelines. First, to ensure our results were not entirely driven by the contrast level of the stimuli, we regressed stimulus contrast level on each trial out of the preprocessed MEG data. Second, to investigate whether differences in prestimulus activity contributed to differences in perceptual visibility (17, 18, 30), we ran our data through the same preprocessing pipeline as above except for two adjustments: removing the baseline correction stage and lengthening the epochs to −450 to 2,000 ms around stimulus presentation. The omission of baseline correction allows the analysis to be sensitive to differences in the prestimulus activity (for example, in the offset or mean amplitude) of trials associated with different awareness ratings which would otherwise be removed by baseline correction (the baseline correction procedure results in each trial's prestimulus window having a mean activity of zero across all time points for each channel, such that our representational similarity analysis [RSA] and decoding analyses would be unable to detect and characterize any prestimulus contribution to visibility codes).

#### Representational similarity analysis

**Fig. 3.**
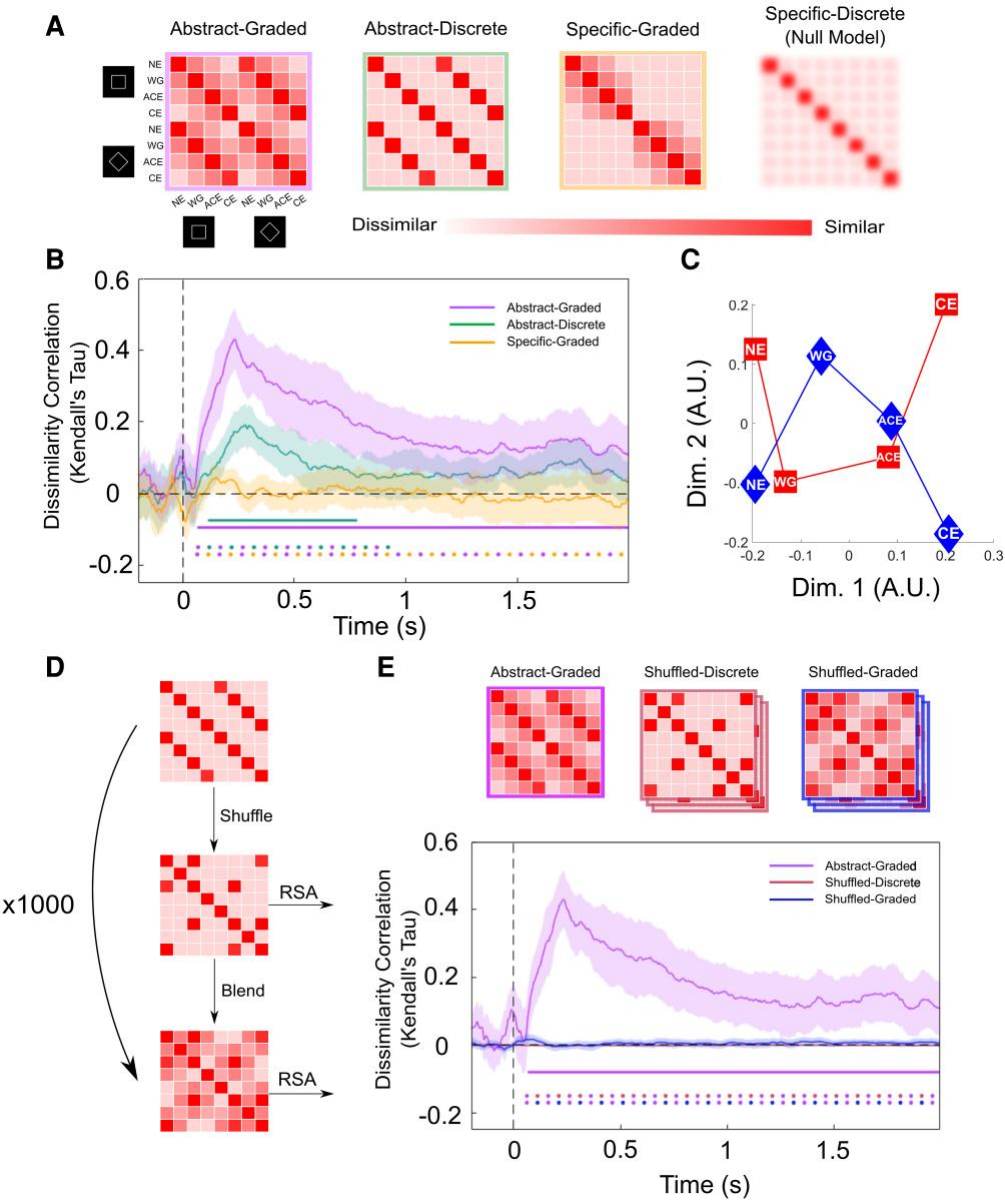
Neural representations of perceptual visibility are abstract and graded. A) From left to right: Abstract-graded model where neural correlates of awareness ratings are independent of perceptual content and follow a graded structure; abstract-independent model where awareness ratings are independent of perceptual content but do not follow a graded structure; specific-graded model where awareness ratings are specific to the perceptual content to which they relate and follow a graded structure; specific-discrete (null hypothesis) model where there is no observable representational structure among awareness ratings (PAS ratings, NE, WG, ACE, CE). B) RSA reveals that the abstract-graded model was the best predictor of the representational structure of neural patterns in whole-brain sensor-level MEG data. Solid horizontal lines represent time points significantly different from 0 for a specific RDM at *P* < 0.05, corrected for multiple comparisons. Horizontal dots denote statistically significant paired comparisons between the different models at *P* < 0.05, corrected for multiple comparisons. We obtained similar findings across occipital (Fig. S1A) and frontal (Fig. S1B) sensors separately, as well as in datasets with stimulus contrast level regressed out (Fig. S2) and without baseline correction (Fig. S5). We also examined the pattern of classifier mistakes during cross-stimulus decoding, again revealing distance-like effects in perceptual visibility decoding (Fig. S4). C) Multidimensional scaling reveals a principal dimension encoding the magnitude of perceptual vividness across square stimuli (red squares) and diamond stimuli (blue diamonds). D) Shuffling and blending procedure. This analysis was performed to control for naturally occurring low-frequency content in neural data. E) Results from both shuffled models reflect the average Kendall's Tau over 1,000 shuffling permutations. Purple, red, and blue lines represent similarity of the abstract-graded, shuffled-discrete, and shuffled-graded models, respectively, with neural data. The shuffled-discrete line varies only slightly from 0 and is thus hard to see. The abstract-graded model is the only model under consideration that significantly predicted the neural data.

RSA allows us to directly compare bespoke hypotheses about the structure of neural data (31). In RSA, hypotheses are expressed as model representational dissimilarity matrices (RDMs), which define the predicted similarity of neural patterns between different conditions according to each hypothesis. In our case, we defined four model RDMs that make different predictions about whether or not awareness ratings generalize over perceptual content, and whether or not each rating leads to a graded activation pattern partially shared by neighboring ratings (Fig. 3A).

In our abstract-graded RDM, we model awareness ratings as being independent of perceptual content (such that ratings of a CE of a square have an identical neural profile to those of a CE of a diamond), as well as being graded in nature (exhibiting a distance effect such that ratings of NE are more similar to those of WG, than of ACE). In the specific-graded RDM, awareness ratings are modeled as being graded in the same way, but they are now represented differently depending on which specific stimulus they are related to. Conversely, the abstract-discrete RDM represents PAS ratings as independent of perceptual content but with no graded structure/distance effects (such that the neural code underpinning a report of NE is equally (dis)similar to the neural code reflecting either a WG or a CE). Finally, the specific-discrete RDM reflects our null model, whereby there is no observable representational similarity structure among conditions, such that neural patterns reflecting one specific awareness rating are equally dissimilar to all other awareness ratings.

RSA involves the comparison of the model RDMs with empirical RDMs constructed from neural data. To do this, we first ran a linear regression on the MEG data with dummy-coded predictors for each of our eight conditions (square trials: NE, WG, ACE, CE; diamond trials: NE, WG, ACE, CE; trial condition coded with a 1, and alternative classes coded with a 0). This resulted in coefficient weights for each condition at each time point and sensor, with the weights representing the neural response per condition, averaged over trials. We then computed the Pearson distance between each pair of condition weights over sensors, resulting in an 8 × 8 neural RDM reflecting the similarity of neural patterns across different awareness ratings and stimulus types (8). Neural RDMs were subsequently smoothed over time via convolution with a 60 ms uniform kernel.

We then compared this neural RDM with our model RDMs. To compare model RDMs with the neural RDM, we correlated the lower triangle of the model and neural RDMs using Kendall's Tau rank correlation (32). We performed this procedure at every time point, resulting in a correlation value at each time point for each model. Importantly, we only correlated the lower triangle of the RDMs, excluding the diagonal to avoid spurious correlations driven by the increased similarity of on-diagonal values compared with off-diagonal values (33). This precluded us from directly testing our specific-discrete model, since it is represented by a uniform RDM, and as such would give identical rank correlation values regardless of the neural RDM it was compared with. However, since this RDM reflects our null model (i.e. that there is no observable representational structure among awareness ratings), this model is implicitly compared with the other model RDMs when we examine whether the correlation of these model RDMs with the neural RDM is >0.

One concern with this approach is that the graded hypotheses may win due to the neural data itself being noisy. In other words, if the neural correlates of ratings are not cleanly dissociable, there will be greater overlap between all ratings in the empirical RDM, including those of close neighbors. To ensure we did not obtain spuriously high similarity with the abstract-graded model in virtue of this model's low-frequency content, we performed a shuffling and blending procedure. This procedure involved shuffling the lower triangle of the abstract-discrete RDM before apportioning neighbors of the four (shuffled) high correlation cells with graded amounts of correlation. The correlation was blurred most for immediate neighbors, and less for diagonal neighbors, matching the format of the graded RDMs (Fig. 3D). We ran this procedure 1,000 times per subject, resulting in 1,000 shuffled-discrete and 1,000 shuffled-graded RDMs. We compared all shuffled-discrete and shuffled-graded RDMs with subjects’ neural data at each time point. Finally, we took the average correlation value for each time point across all permutations such that we had a Kendall's Tau value for both the shuffled-discrete and shuffled-graded RDMs across time per subject. Through this approach, we were able to compare neural data with RDMs that shared no representational similarity with our abstract-graded RDM while controlling for differences in variance and frequency profile.

#### Within-subject multivariate decoding analysis

To support and extend conclusions drawn from our RSAs, we ran an exploratory analysis using temporal generalization methods (34) to identify content-invariant and graded representations of awareness ratings while also investigating the stability of these representations over time. In this procedure, a separate classifier is trained on each time point (from 200 ms prestimulus to 2,000 ms poststimulus) and tested on all other time points. This method results in a time-by-time decoding accuracy matrix, indicating the extent to which neural representations are stable over time. The above-chance decoding at a particular point in the decoding matrix indicates that neural representations present at the training and testing time points are similar, while chance decoding indicates the representations have changed.

We ran the above temporal generalization analysis using both a within-condition and a cross-condition decoding procedure. Within-condition decoding involved training and testing the decoder to classify PAS ratings on trials from one stimulus type (either squares or diamonds). In cross-condition decoding, we trained on trials from one stimulus type and tested on trials from the other (e.g. trained on square trials and tested on diamond trials, and vice versa). In both cases, we used a 5-fold cross-validation scheme, with a balanced number of trials per class within each fold. Cross-condition decoding, where a classifier trained to decode multivariate neural patterns in one class of stimuli is tested on an unseen class of stimuli, offers an empirical test of whether the neural patterns associated with each class share a similar neural code across conditions (35–37). This analysis, therefore, complements the RDM analysis in being able to test for content-invariant perceptual visibility codes, while also providing information about their stability over time. We performed all multiclass decoding analyses with a multiclass linear discriminant analysis (LDA) decoder using the MVPA-light toolbox (38) with FieldTrip. Each of the four PAS ratings served as classes for the decoder to classify trials into. We used L1-regularization of the covariance matrix, with the shrinkage parameter calculated automatically using the Ledoit–Wolf formula within each training fold (39).

It is important to note that cross-validation is not technically necessary during cross-condition decoding because the test data are never seen by the classifier during training, so there is no risk of overfitting. However, we employed a cross-validation scheme for all our decoders so that differences in their performance would not be due to differences in training procedures (e.g. number of trials in the training or test set). Data were smoothed over 7 samples (28 ms), and classification analysis was run at individual time points throughout the whole trial to characterize the temporal dynamics of the representations (−200 to 2,000 ms poststimulus).

#### Stimulus decoding

One difficulty with interpreting a content-invariant representation of perceptual visibility is that it may reflect a lack of power to detect content-specific differences between conditions (e.g. square vs. diamond). To control for this possibility, we sought to ensure that the resolution of our data was sufficiently fine-grained to pick up differences in the neural encoding of different stimuli. To do this, we applied a binary decoding procedure using a binary LDA decoder with the same classification parameters as above. In this analysis, the two stimulus types (squares and diamonds) were used as classes for the decoder to classify trials into. For this analysis, we grouped trials into low-visibility (NE and WG) and high-visibility (ACE and CE) trials to ensure sufficient power, performing the decoding analysis separately in each group. Once again, data were smoothed over 7 samples (28 ms) and analyzed on individual time points throughout the whole trial (−200 to 2,000 ms poststimulus).

#### Statistical inference

To determine whether our RSA and decoding results were statistically significant, we used cluster-based permutation testing (40) with 1,000 permutations. For RSA, within each permutation, we flipped the sign of each ranked correlation value at each time point for each participant and performed a one-sample *t* test against 0. Resulting *t*-values associated with a *P*-value <0.05 were used to form clusters across the single time dimension. For each cluster, an associated cluster statistic was computed, the largest of which was stored per permutation to build a group-level null distribution. The cluster statistic computed from our observed data was then compared with this chance distribution to determine statistical significance with an alpha level of 0.05. This procedure controls for the multiple comparisons problem by only performing one comparison at the inference stage and specifically tests the null hypothesis that the observed data are exchangeable with data from the permuted (null) distribution (40). We used the same cluster-based permutation procedure to compare how well different model RDMs predicted the neural data. In this case, we performed paired comparisons, where ranked correlation values per RDM were randomly swapped within subjects per permutation to build up a group-level null distribution.

For decoding results, we used the same cluster-forming parameters, but this time we randomly flipped the sign of individual subjects’ accuracy scores per permutation to build up a group-level null distribution. Additionally, we formed clusters over both time dimensions of the temporal generalization matrices. We used the same cluster-based permutation procedure to compare performance between cross-condition and within-condition decoders.

It is important to note that our cluster-based permutation testing procedure does not allow for inference as to the exact time points at which neural representations come into existence. This is because the algorithm does not consider individual time points at the statistical inference stage, since at this point, it only relies on cluster statistics, which encompass multiple time points (41). Still, as we are not interested in the precise onset of content-invariant representations of awareness ratings but rather their general temporal profile, this method is sufficient for our purposes.

### fMRI experiment

To help localize representations of perceptual visibility in the brain, we reanalyzed a previously collected fMRI dataset (26). It is worth noting that, while source-space decoding in MEG is certainly possible (25, 42), fMRI is much better suited to answering this question at a fine spatial scale, especially as we wish to compare the (potentially fine-grained) differences and similarities in regional activity covarying with perceptual content and/or visibility.

#### Participants

Thirty-seven participants took part in the study. The study was approved by the local ethics committee (CMO Arnhem-Nijmegen), and all participants gave informed written consent prior to participating. Eight participants were excluded from our analyses. One was excluded because they quit the experiment early, and another because they failed to follow task instructions. The final six subjects were excluded because they did not have at least 10 trials in each visibility rating class after our grouping procedure (see the Within-subject multivariate searchlight decoding analysis section). Twenty-nine subjects were thus included in our final analyses (mean age = 25.35; SD = 6.31).

#### Stimuli

The stimuli used were taken from the POPORO stimulus dataset (43). The stimuli selected were a rooster, a fish, a watering can, and a football (Fig. 2D), and were selected based on familiarity and visual difference to maximize classification performance as well as both accuracy and visibility scores calculated in a pilot experiment run by Dijkstra et al. (26). The mask was created by randomly scrambling the pixel values of all stimuli combined (Fig. 2C).

#### Experimental design and statistical analyses

The experiment consisted of two tasks: a perception task and an imagery task. Each of these tasks was executed in interleaved blocks and was counterbalanced across participants. Our reanalysis used data only from the perception task, and, thus, we omit details of the imagery component of the study. The perception task ran as follows. A stimulus was presented for 17 ms, followed by a backward mask for 400 ms. Participants then indicated whether the stimulus was animate or inanimate and rated the visibility of the stimulus on a scale from 1 (not visible at all) to 4 (perfectly clear). Button response mappings were counterbalanced across trials. The task was made up of visible and invisible trials. The difference between these trials was the length of the interstimulus interval (ISI) between the stimulus and the mask. In the visible trials, the ISI was 66 ms, and in the invisible trials, the ISI was 0 ms. In the present study, we only analyzed data from invisible trials (Fig. 2C) because these were associated with the variation in the visibility ratings that we are interested in. Choosing to focus on a single ISI also means that differences in visibility ratings were not driven by differences in stimulus presentation characteristics. There were 184 trials in total, with 46 repetitions per stimulus divided over 4 blocks. More detailed information regarding the study protocol can be found in Dijkstra et al. (26).

#### Acquisition

fMRI data were recorded on a Siemens 3T Skyra scanner with a Multiband 6 sequence (repetition time [TR]: 1 s; voxel size: 2 × 2 × 2 mm; echo time [TE]: 34 ms) and a 32-channel head coil. The tilt of each participant's field of view was controlled using Siemens AutoAlign Head software, such that each participant had the same tilt relative to their head position. T_1_-weighted structural images (MPRAGE; voxel size: 1 × 1 × 1 mm; TR: 2.3 s) were also acquired for each participant.

#### Preprocessing

The data were preprocessed using SPM12 (RRID: SCR_007037). Motion correction (realignment) was performed on all functional imaging data before coregistration with the T_1_ structural scan. The scans were then normalized to MNI space using DARTEL normalization and smoothed with a 6-mm Gaussian kernel, which has been shown to improve group-level decoding accuracy (44–46). Slow signal drift was removed using a high pass filter of 128 s.

#### General linear model

Coefficient weights were estimated per trial with a general linear model that contained a separate regressor for each trial at the onset of the stimulus convolved with the canonical HRF. Alongside nuisance regressors (average WM and CFG signals and motion parameters), the screen onset and button press of both the animacy and visibility responses were included as regressors, as well as a constant value per run to control for changes in mean signal amplitude across runs.

#### Within-subject multivariate searchlight decoding analysis

For decoding our fMRI data, we binarized the visibility ratings into low- and high-visibility classes. This is because, in contrast to the MEG experiment, visibility was not staircased per participant, leading to a large number of participants failing to have enough trials at each of the four visibility ratings in both animate and inanimate trials. Because training a decoder on such a small number of trials would yield unreliable and noisy results, trials were, therefore, sorted into low- and high-visibility classes on a subject-by-subject basis prior to analysis. This was performed as follows: The median visibility rating (from 1 to 4) was extracted from each subject, and trials with a lower visibility rating than the median were classed as low-visibility trials, and those with visibility ratings equal to or greater than the median were classed as high-visibility trials. This procedure allowed us to control for the fact that different subjects had different distributions of visibility ratings, such that the lower 1 and 2 ratings did not always correspond to low-visibility trials, and likewise the higher 3 and 4 ratings did not always correspond to high-visibility trials. For instance, 1 subject may have used visibility ratings 2 and 3 in around 50% of trials, rating 4 on the other 50%, and not used rating 1 at all. In this case, we would label ratings 2 and 3 as low visibility, and rating 4 as high visibility.

Trials were next grouped according to whether they contained an animate or inanimate stimulus. For each participant, if there were <10 trials in either the low- or high-visibility class for either the animate or inanimate trial, the participant was removed. This was the case for six participants. The mean number of trials per condition following this procedure was as follows (numbers in brackets denote the SD): animate-high visibility: 63.48 (11.34); animate-low visibility: 25.31 (10.38); inanimate-high visibility: 61.10 (12.24); inanimate-low visibility: 28.86 (11.48).

We used an LDA classifier on the beta estimates per trial to decode low- and high-visibility ratings within and across animate/inanimate stimulus conditions. Cross-condition decoding was performed by training the LDA classifier on low- vs. high-visibility ratings in animate trials and then testing it on low- vs. high-visibility ratings in inanimate trials, and vice versa. Cross-condition decoding was performed with the same logic as in our MEG analysis: if we train a classifier to decode visibility ratings in animate trials and use this classifier to successfully decode visibility ratings in inanimate trials, we can conclude the representations of visibility ratings are similar across different perceptual content. Once again, we also performed within-condition decoding, where the classifier was trained on low vs. high ratings in one condition (e.g. animate trials), and tested on trials in the same condition to allow a direct comparison of within- and across-condition decoding performance. This comparison allowed us to determine where content-specific representations of perceptual visibility may exist in the brain. Decoding was performed with a 5-fold cross-validation scheme using L1 regularization with a shrinkage parameter of 0.2, and, similar to the MEG analysis, cross-validation was used for both within-condition and cross-condition decoding. Trials were down-sampled prior to decoding, such that there were an equal number of low- and high-visibility trials in each fold. To ensure that our data were sensitive enough to show content-specific codes, we additionally ran a similar analysis that sought to decode stimulus content (animate or inanimate) rather than visibility. This analysis was similar in structure except the classifier was trained to decode animate vs. inanimate trials rather than visibility level.

Decoding was performed using a searchlight method. Searchlights had a radius of 4 voxels (257 voxels per searchlight). As such, at every searchlight, the classifier was trained on 257 features (1 beta estimate for each voxel in the searchlight) for each trial in every fold. The searchlights moved through the brain according to the center voxel, meaning that each voxel was entered into multiple searchlights. After decoding in each searchlight, the accuracy of the classifier was averaged across folds, and this value was stored at the center of the searchlight to produce a brain map of decoding accuracy.

#### Stimulus decoding in fMRI regions of interest

As in the MEG analysis, we again wished to establish that findings of content-invariant awareness representations were not due to an inability to decode content itself. We tested our ability to decode perceptual content within two regions of interests (ROIs) with successful visibility decoding from our searchlight results. To do this, we created two masks, one visual and one frontal, and then selected the 200 voxels within this mask that had the highest mean visibility decoding accuracy averaged across all four decoding directions (within animate; within inanimate; train animate-test inanimate; train inanimate-test animate). For the frontal mask, we used a connectivity-based parcellation of the orbitofrontal cingulate cortex that spanned frontal regions with successful visibility decoding. These were regions 8m (*x, y, z* peak voxel coordinates per hemisphere*—*LH: −14.6, 33.8, 43.3; RH: 13.5, 32.3, 44) and 32d (LH: −8.7, 37.5, 23.4; RH: 12.7, 40.4, 17.5) (47). Our visual mask spanned an area with successful visibility decoding in occipital regions VO1 (LH: −27.1, −70.9, −11.3; RH: 27.5, −69.5, −10.6), VO2 (LH: −25.6, −64.3, −10.6; RH: 26.7, −59.9, −9.1), PHC1 (LH: −27.1, −54, −8.3; RH: 28.3, −53.2, −8.3), and PHC2 (LH: −28.6, −45.9, −8.3; RH: 29, −43.7, −9.8) (48). The coordinates for the clusters obtained within each ROI are summarized in Table S1. Using the 200 ROI voxels as features, we decoded animate (rooster and fish) vs. inanimate (watering can and football) stimuli in low- and high-visibility trials separately using the same 5-fold cross-validation procedure and LDA parameters as above, down-sampling trials prior to decoding to ensure an equal number of animate and inanimate trials in each fold.

#### Group-level statistical inference

Distributions of accuracy values from the classification of fMRI data are often non-Gaussian and asymmetric around the chance level. This means that parametric statistical comparisons, such as *t* tests against chance decoding (50%), are unable to provide valid tests of whether group-level accuracy values are significant (49). Therefore, to determine where classifiers had performed significantly above chance, we compared mean performance across all participants with a null distribution created by first permuting the class labels 25 times prior to decoding per participant and then using bootstrapping to form a group-level null distribution of 10,000 bootstrapping samples (49). We did this separately for each decoding direction (within: train and test on animate; train and test on inanimate; cross: train on animate, test on inanimate; train on inanimate, test on animate). To perform statistical inference on an average cross-decoding map created by averaging the two cross-condition decoding directions, this average map was compared with a group-level null distribution formed by averaging the two null distributions created for the two separate maps. To compare within-condition and cross-condition classification performances, a group-level null distribution was formed by taking the difference between cross and within decoding scores throughout the bootstrapping procedure. To control for multiple comparisons in the searchlight analysis, the resulting *P*-values were subsequently corrected for multiple comparisons with a false discovery rate of 0.01.

## Results

### Representational structure of perceptual visibility in whole-brain MEG data

We used RSA to test whether perceptual visibility levels (PAS ratings) correlated with MEG activity patterns independently of perceptual content (abstract RDMs) or together with perceptual content (specific RDMs). We additionally tested whether neural activity patterns covaried with visibility levels in a graded or discrete manner (Fig. 3A). A model instantiating graded and abstract representations of awareness ratings significantly predicted the neural data throughout most of the poststimulus period (purple line; Fig. 3B). In contrast, a model with an abstract but discrete representational structure was able to predict the neural data only in an early phase of the trial between ∼100 and 500 ms after stimulus onset (green line). Paired comparisons between these two models showed that the abstract-graded model was significantly better at predicting the neural data than the abstract-discrete model throughout the majority of the trial (purple and green dots). The specific-graded model did not significantly predict the neural data at any point during the trial (gold line), and likewise the abstract-graded model was found to be significantly better at predicting the neural data than the specific-graded model in a direct comparison (purple and yellow dots), indicating that an abstract model of awareness ratings better described their neural representation. In line with this, multidimensional scaling of awareness ratings revealed a principal dimension encoding vividness that was shared by both square and triangle stimuli (Fig. 3C). To assess the spatial distribution of abstract-graded signals across sensors, we repeated the analysis for frontal and occipital sensors separately (following Ref. (50)), finding similar results in each case (Fig. S1). These results indicate that neural correlates of perceptual visibility generalize over perceptual content and exhibit distance effects, indicative of neural populations tuned to specific degrees of visibility with overlapping tuning curves.

To ensure that the neural data did not exhibit spuriously high similarity with the abstract-graded model in virtue of its increased variance and reduced frequency when compared with the abstract-discrete and specific models, we performed a shuffling and blending control analysis (Fig. 3D). This procedure revealed no significant prediction of the neural data for either the shuffled-discrete or shuffled-graded RDMs (Fig. 3E). As such, RDMs with frequency and variance profiles matching those of the abstract-graded RDM, but without any relationship with awareness ratings, were not able to significantly predict neural data, in contrast to the abstract-graded model that captures the graded and content-invariant structure of awareness ratings. To additionally control for the possible influence of stimulus contrast on our RDM results, we confirmed that similar results were obtained when regressing out the linear component of contrast (Fig. S2). It is possible that nonlinear or multivariate effects of contrast may have still driven some of our findings. Indeed, while we see a clear linear trend from NE to CE across the first dimension in the original data, this dimension is somewhat compressed following the removal of the linear component of stimulus contrast. Along this compressed dimension, higher ends of the scale are represented more similarly than those at the lower end. This is potentially in line with a Weber scaling law in the neural representation of perceptual vividness, as also found for other magnitude codes (e.g. the “size effect” in numerical cognition), and also hints at a role for stimulus contrast in driving some of the difference between CE and ACE in the original analysis. However, even after removing the potentially confounding effects of stimulus contrast, the difference in perceptual vividness among NE, WG, and ACE/CE is clearly distinguished in Fig. S3.

To further characterize the graded representational structure of perceptual visibility, we computed confusion matrices between each rating and its neighbors. By plotting the proportion of predictions for each awareness rating made by the multiclass classifier separately for trials of each rating, we can visualize when our decoder makes mistakes, and which PAS ratings it most often confuses (Fig. S4). These confusion plots confirm the distance effects identified with the RSA model comparison, in which neighboring PAS ratings are most often confused with each other by the classifier, and more distant ratings less so, suggesting that visibility is represented in a graded, ordinal manner.

Finally, we asked whether our model RDMs could also predict prestimulus neural activity. If a graded, abstract structure for perceptual visibility is already evident prior to stimulus presentation, this would be indicative of trial-to-trial fluctuations in attention or arousal contributing to our ability to decode content-invariant visibility signals. Interpreting (a lack of) prestimulus decoding from our previous RSAs is confounded by the baseline correction procedure applied during preprocessing. To address this issue, we reran our analysis on data that had not been baseline corrected. We found that prestimulus activity was not captured by any of the candidate RDMs and that stimulus-triggered responses continued to show the same graded/abstract pattern of results as in our initial analysis (Fig. S5). Together, these results indicate that pretrial fluctuations in attention and/or arousal are unlikely to drive our results.

### Temporal profile of perceptual visibility codes

**Fig. 4.**
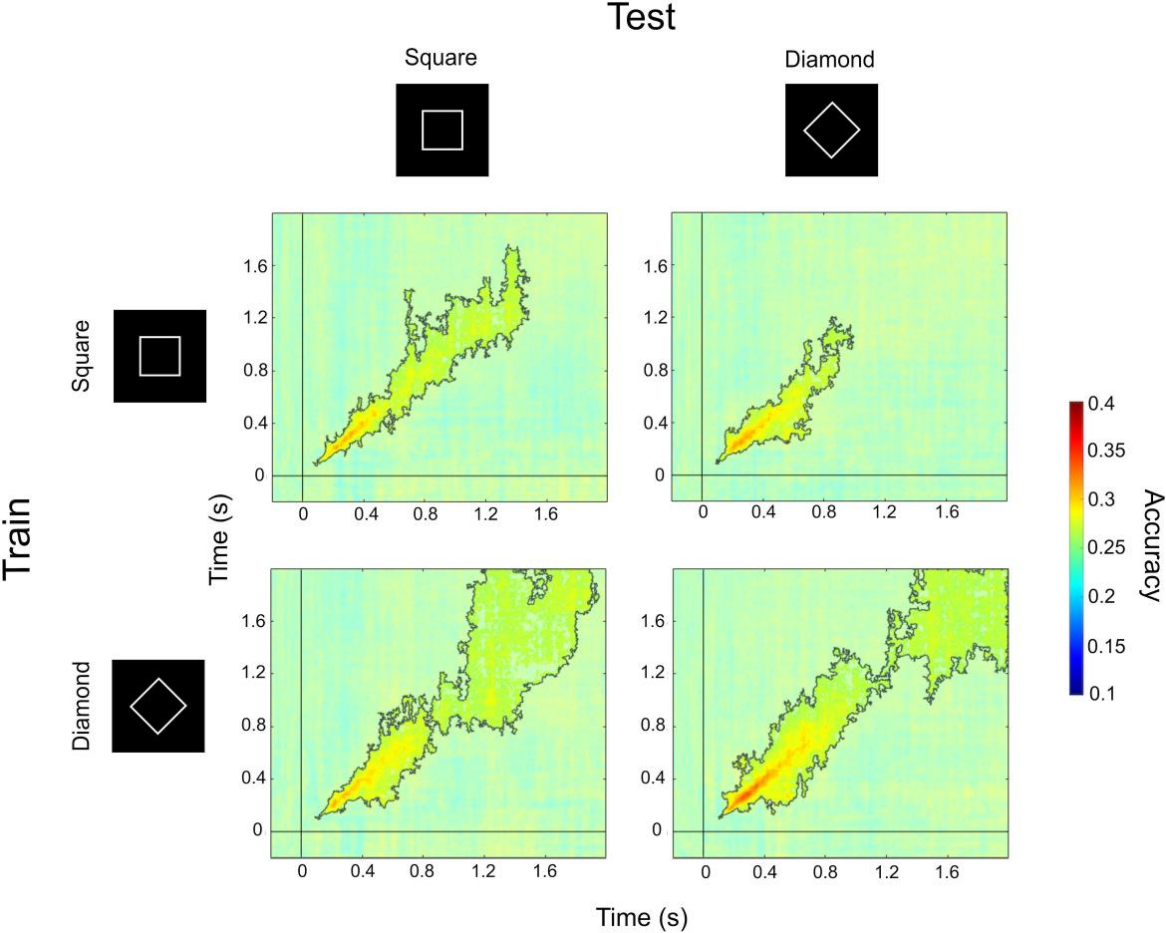
Abstract representations of perceptual visibility evolve rapidly over time. Main figure: Temporal generalization results for the classification of PAS ratings from MEG data (4 PAS responses; chance = 0.25). For each row, statistical comparisons between the two columns showed no significant differences in decoding accuracy between within- and cross-condition decoding. Nontranslucent regions within solid lines highlight above-chance decoding, as revealed by cluster-based permutation tests. We replicated these findings in nonbaseline-corrected data (Fig. S6).

Next, we performed a temporal generalization analysis to further unpack the content-invariant nature of neural signatures of perceptual visibility and to characterize how and whether their patterns change from time point to time point. Off-diagonal panels in Fig. 4 (top right and bottom left) depict temporal generalization matrices for both directions of cross-condition decoding (top right: train on squares test on diamonds; bottom left: train on diamonds test on squares). Within these panels, above-chance decoding on the major diagonal indicates that representations of visibility begin to show content-invariance from just after stimulus onset up until the moment of report. Contrasting cross-condition decoding with within-condition decoding resulted in no significant differences in decoding accuracy for either comparison (train on squares, test on diamonds vs. within squares: all *P* > 0.89; train on diamonds, test on squares vs. within diamonds: all *P* > 0.4). In other words, we did not find any evidence that there was content-specific visibility information available over and above content-invariant information. Furthermore, the lack of off-diagonal decoding in each temporal generalization matrix indicates that the format of content-invariant neural signatures of visibility changes rapidly over time.

We again replicated this analysis in a dataset that had not undergone baseline correction to test whether activity contributing to participants’ awareness ratings could be decoded prior to stimulus presentation. In line with our RSA on this dataset, we could not decode awareness ratings prior to stimulus presentation when the data had not been baseline corrected (Fig. S6).

### Content-invariant representations of visibility are found across visual, parietal, and frontal cortex

**Fig. 5.**
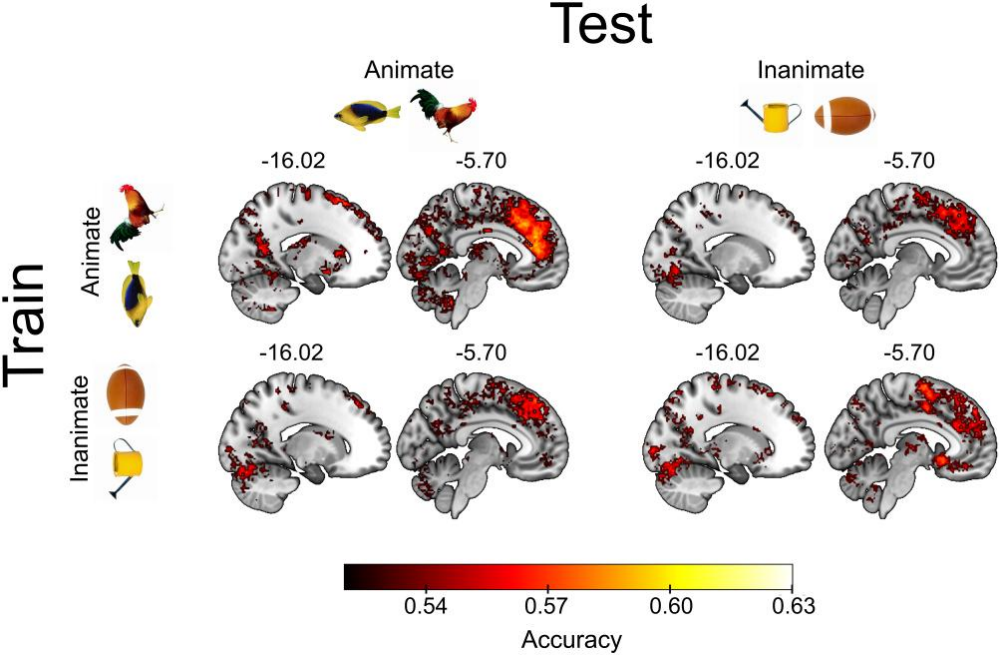
Abstract representations of perceptual visibility are found across visual, parietal, and frontal cortex. Searchlight decoding in fMRI data revealed significantly above-chance accuracy in both cross-condition (off-diagonal cells of matrix) and within-condition (on-diagonal cells) in decoding of visibility ratings. Clusters of successful cross-condition decoding were found across the frontal, parietal, and visual cortex. Our statistical comparison of cross and within-condition decoding accuracy (comparing the on- and off-diagonal statistical maps) revealed no significant differences anywhere in the brain. Significance was assessed at *P* < 0.05, corrected for multiple comparisons with a false discovery rate of 0.01. Clusters are reported in Table S2.

To localize brain regions supporting content-invariant representations of perceptual visibility, we reanalyzed an existing fMRI dataset (26). We used a searchlight approach to identify brain regions that represent perceptual visibility in an abstract manner. Both cross-condition and within-condition decoding resulted in above-chance accuracy in a number of regions across the visual, parietal, and frontal cortex (Fig. 5). To assess whether these representations of perceptual visibility were stimulus dependent, we compared cross-condition decoding to within-condition decoding in both animate and inanimate trials (training on animate trials vs. within animate trials; training on inanimate trials vs. within inanimate trials), and found no significant differences. In other words, we could find no evidence that stimulus-specific visibility information was present over and above stimulus-invariant visibility information. See Table S2 for details of the clusters found to be significantly above chance in both cross-condition and within-condition decoding analyses.

### Stimulus content can be decoded from both MEG and fMRI data

**Fig. 6.**
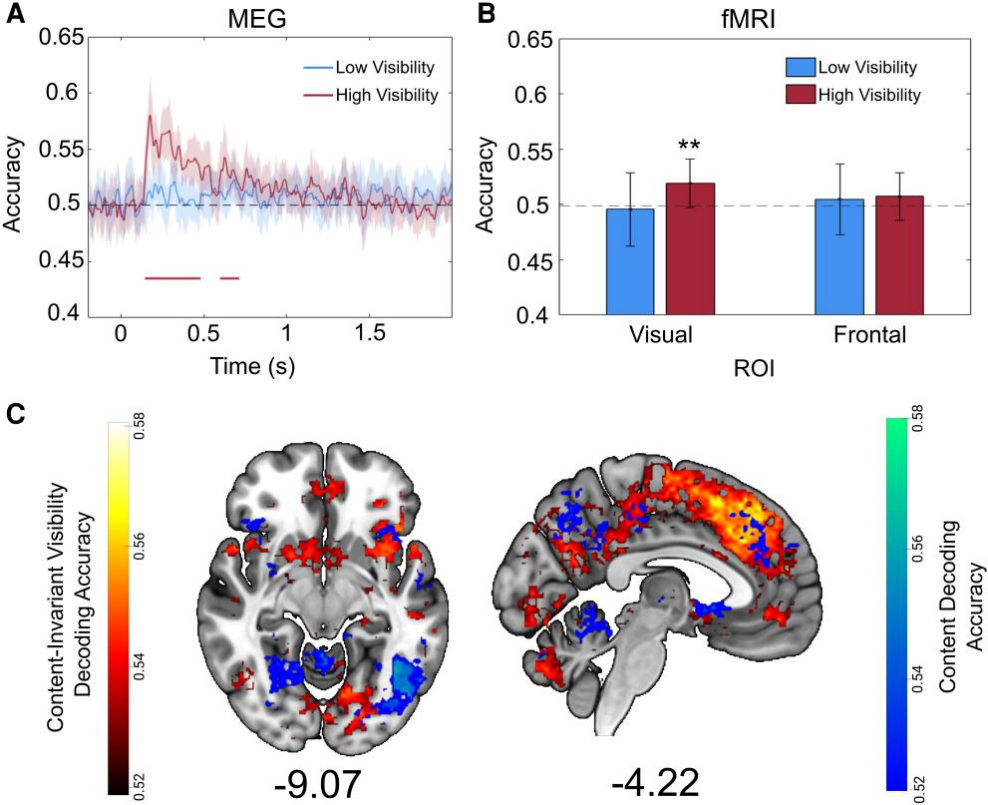
Perceptual content can be decoded in high-visibility trials and shows distinct representations to visibility. A) Decoding of perceptual content on each trial (squares or diamonds) from participants’ whole-brain sensor-level MEG data for low-visibility (NE and WG) and high-visibility (ACE and CE) trials separately. Successful decoding was possible in high-visibility trials up to ∼700 ms poststimulus onset. Lines are smoothed using a Gaussian-weighted moving average with a window of 20 ms. Shaded area denotes 95% CIs. The solid horizontal line reflects above-chance decoding, as revealed by cluster-based permutation tests. B) Decoding of perceptual content on each trial (animate or inanimate) from participants’ fMRI data for low- and high-visibility trials separately. Decoding was successful in a visual ROI in high but not low-visibility trials, and unsuccessful in a frontal ROI. Asterisks denote significance at *P* < 0.01. Error bars illustrate 95% CIs. C) Searchlight decoding accuracy for content decoding in high visibility trials (blue) and for content-invariant visibility decoding (red). Clusters illustrate areas where content or content-invariant visibility could be decoded significantly above chance. Content-invariant representations of visibility were more widespread than content representations and extended into the prefrontal cortex, whereas both content and visibility could be decoded in distinct locations of the visual cortex. Significance was assessed at *P* < 0.05, corrected for multiple comparisons with an FDR of 0.01. Clusters are reported in Table S3.

We next considered the possibility that a content-invariant neural signature of visibility may be obtained because of the insufficient sensitivity to perceptual content in our dataset. To address this, we sought to decode stimulus identity, rather than visibility level. Stimulus decoding was above chance in both datasets for high-visibility trials. In the MEG data, we were able to decode stimulus identity (square vs. diamond) in trials in which participants used the upper two PAS ratings (ACE/CE), but not when participants used the lower two PAS ratings (NE/WG; Fig. 6A). Similarly, in the fMRI data, the decoding of animate vs. inanimate stimuli was significantly above chance in a visual cortical ROI during trials reported as high visibility (mean accuracy = 0.52; *P* = 0.007) but not in trials reported as low visibility (mean accuracy = 0.496; *P* = 0.655; Fig. 6B). It was not possible to decode stimulus content from a frontal cortical ROI in either low-visibility (mean accuracy = 0.5; *P* = 0.406) or high-visibility trials (mean accuracy = 0.507; *P* = 0.159). Together, these analyses indicate that stimulus content could be reliably decoded in posterior visual regions.

### Stimulus content and visibility are encoded in dissociable brain regions

To further probe the relationship between neural signatures of content and visibility, we ran a searchlight decoding procedure to decode animate vs. inanimate stimuli in our fMRI data. Since content could only be decoded in high-visibility trials in our ROI analysis (Fig. 6B), we restricted the analysis to these trials. We then compared the overlap between the content-decoding searchlight and the content-invariant visibility searchlight maps. To do this, we computed mean content cross-decoding accuracy averaged over the two cross-decoding directions (train on animate, test on inanimate; train on inanimate, test on animate) prior to group-level inference (see Materials and methods).

Overall, there was a minimal overlap between representations of content and visibility (Fig. 6C). Despite overlapping clusters being obtained in the superior and inferior lateral occipital cortex (see Table S3 for a full list of individual and overlapping clusters), clear anatomical distinctions in occipital regions can be seen between representations of stimulus content and visibility, with the former being decoded from more lateral regions of the occipital cortex, while the latter was decoded closer to the medial surface (Fig. 6C and Table S3). Fewer clusters of above-chance stimulus content decoding were found in frontal regions, whereas content-invariant representations of visibility were more abundant in these areas (51). Distinct decoding patterns for content and visibility representations further strengthen the notion that content-invariant representations of visibility exist partly independently of perceptual content, even in regions typically associated with the encoding of stimulus content such as the visual cortex (52, 53).

## Discussion

In this study, we asked whether perceptual vividness covaries with neural activity patterns in a content-specific and/or content-invariant manner. By applying multivariate analyses to MEG and fMRI datasets in which participants rated their awareness of visual stimuli, we found that the vividness of experience is represented in a similar way across different stimulus contents and exhibits signatures of an ordered and graded magnitude code. Furthermore, neural representations of perceptual vividness were found to change rapidly over time and were localized to the visual, parietal, and frontal cortices.

The identification of content-invariant representations of perceptual vividness (Fig. 3) is in line with recent work highlighting a dissociation between neural correlates of awareness and perceptual content. For example, Sanchez et al. (19) found neural patterns that indicated whether an individual was aware of a stimulus or not, irrespective of which sensory modality it was presented in. Likewise, Mazor et al. (54) reported that, while stimulus identity was best decoded from occipital regions, perceptual visibility (stimulus presence vs. absence) could be effectively decoded from a wider range of areas, including the parietal and frontal cortex. Notably, a recent study also found that graded changes in perceptual vividness could be reliably decoded from the prefrontal cortex, even in the absence of a report, consistent with a contribution to the vividness of experience (51). While we do not claim that representations of vividness are solely content-invariant, we build on these findings by showing that neural signals underlying graded awareness ratings—ranging from the absence of an experience of particular content, to a clear and vivid experience—exhibit a content-invariant neural signature.

Content-invariant representations of vividness may also provide a new understanding of the mechanisms supporting intensity-matching in psychophysical tasks. For example, studies of cross-modal intensity matching have demonstrated that subjects can reliably match intensities across sensory domains (55–57), and even provide some evidence for absolute equivalences between intensities in different modalities (55). Success in such tasks could be mediated by some form of common currency for intensity that is modality-invariant, and our findings offer a potential neural framework within which to explain this capacity. Specifically, if the intensity of an experience is mapped onto a low-dimensional and content-invariant neural code for vividness, it should be possible to leverage this representation to reliably match the intensity of stimuli across sensory modalities. This is the essence of “mapping theory” (58) and could be directly tested by combining intensity-matching psychophysical methods with neuroimaging to examine the degree to which psychophysical estimates of cross-modal magnitudes rely on the same low-dimensional neural manifolds associated with vividness observed here.

Although we find evidence for content-invariant signals underlying perceptual vividness, the mechanism by which these signals influence vividness remains to be determined. One candidate mechanism may be the top-down modulation of content-specific representations, driven by content-invariant attention signals. For example, fluctuations in the (content-invariant) degree of attention may increase the perceived contrast of stimuli (59, 60), perhaps through the modulation of content-specific neuronal responses. In line with this model, there may be multiple components to a neural representation of perceptual vividness: content-invariant signals that are associated with the degree of attention or other domain-general factors, and content-specific representations modulated by such attentional signals. Such a model neatly exemplifies how content-invariant and content-specific neural signatures may together contribute to the subjective experience of perceptual vividness. Indeed, on this view, content-specific modulations may be subtle compared with changes in abstract representations determining the degree of attention, which could in turn explain why our content-specific vividness model did not provide a good fit to the neural data.

Our finding that neural representations of awareness ratings display a distance effect (Figs. 3 and S4) is suggestive of perceptual vividness relying on similar schemes to those encoding magnitude in other domains such as number. Specifically, our results are consistent with the possibility that distributed populations of neurons are tuned to specific phenomenal magnitudes, in the same way that specific populations of neurons are sensitive to certain numerical magnitudes (61–63). Such a prediction could be tested through repetition suppression experiments (63), and/or by collecting single-unit recordings from human patients while they provide subjective awareness ratings (64). A variety of analog magnitudes have been shown to rely on common magnitude representations (8, 65, 66), prompting a hypothesis that domain-general representations are responsible for encoding low-dimensional quantities in the brain (9, 10). Therefore, an intriguing possibility is that perceptual vividness is supported by similar domain-general magnitude codes. Future work could explore this hypothesis by assessing whether representations of vividness share neural resources with other analog magnitude codes, such as those for reward or number (8).

The existence of stimulus-independent representations of perceptual vividness in the visual cortical areas (Fig. 5) was unexpected, since these areas have been shown to distinguish stimulus features rather than subjective vividness in previous studies (52, 53). One concern is that neural representations of vividness ratings as revealed by decoding analyses may look similar across stimuli if content-specific information encoded in separate neural populations is treated as belonging to the same population (i.e. within the same voxel). Successful cross-stimulus decoding of vividness ratings could then occur by way of decoding the amplitude of (content-specific) neural responses in these voxels (23, 24). As a step toward addressing this concern, we show that stimulus-specific decoding remains possible specifically in visual areas on high (but not low)-visibility trials (Fig. 5A and B), suggesting that the content-invariant nature of perceptual vividness signals in this region is not due to a lack of power to detect stimulus-specific effects. Moreover, we show anatomical distinctions between content and visibility encoding (Fig. 6C), again indicating that the unexpected above-chance decoding of visibility in the visual cortex is unlikely to be an artifact of a failure to detect content-specific representations.

Another possibility is that content-invariant signals of perceptual vividness in visual cortex reflect prestimulus activations that have been shown to contribute to participants’ awareness level in previous studies (17). Here, we could not identify prestimulus contributions to visibility codes in our MEG data (Fig. S6), supporting the hypothesis that the content-invariant and graded representations we report here are largely stimulus-triggered. As such, our results suggest that the content-invariant signals related to awareness level in the current data are partly distinct from those reported by Podvalny et al. in temporal profile. In any case, it is worth noting that fluctuations in (pre- or poststimulus) attention and arousal affecting the intensity of experience (as well as other psychological factors such as emotional state or motivation) may provide domain-general sources of perceptual vividness signals.

By applying temporal generalization analysis to our MEG data, we were able to reveal the dynamics of vividness representations over time. This analysis indicated that neural patterns covarying with perceptual vividness are unstable, changing during the course of a trial (Fig. 4), consistent with a sequence of different neural populations correlating with awareness level over time (34). Given that we find that vividness is tracked across a variety of cortical regions, such a rapidly changing temporal profile may reflect dynamic message passing between distinct neural populations, consistent with the reverberation of predictions and prediction errors in hierarchical generative models. Future work to directly test this hypothesis could leverage informational connectivity analyses (67) to determine the direction of information flow across interacting brain regions, or use RSA to combine MEG/EEG and fMRI data collected using the same task and stimuli (68).

In summary, we show that perceptual vividness covaries with content-invariant neural representations that exhibit graded distance effects similar to those observed for analog magnitude codes in other cognitive domains. These representations are spatially distributed and rapidly evolve over time, consistent with the flow of awareness-related information across the visual, parietal, and frontal cortices. This pattern of results adds to growing evidence for a content-invariant neural component contributing to the strength of conscious experience.

## Supplementary Material

pgae061_Supplementary_Data

## Data Availability

All codes are available at the following GitHub Repository: https://github.com/benjybarnett/abstract-awareness. fMRI data are accessible at https://doi.org/10.34973/j9yn-q419. MEG data were collected prior to General Data Protection Regulations (GDPR). Participants were therefore not asked for their consent to make their data public. As such, according to GDPR stipulations (which now apply to the data), the MEG data cannot be made publicly available. Data may, however, be shared privately upon request.

## Preprints

This manuscript was posted on a preprint: https://www.biorxiv.org/content/10.1101/2022.11.30.518510v3.
