# Supplementary material for: Identifying content-invariant neural signatures of perceptual vividness
Source: PNAS Nexus. 2024 Feb 14;3(2):pgae061. doi: 10.1093/pnasnexus/pgae061 (PMC10898512; doi:10.1093/pnasnexus/pgae061)
Supplement: pgae061_Supplementary_Data [file pgae061_supplementary_data.zip › PNASNEXUS-PNASNEXUS-2023-00872RR-s01.docx]

**Supplemental Information**


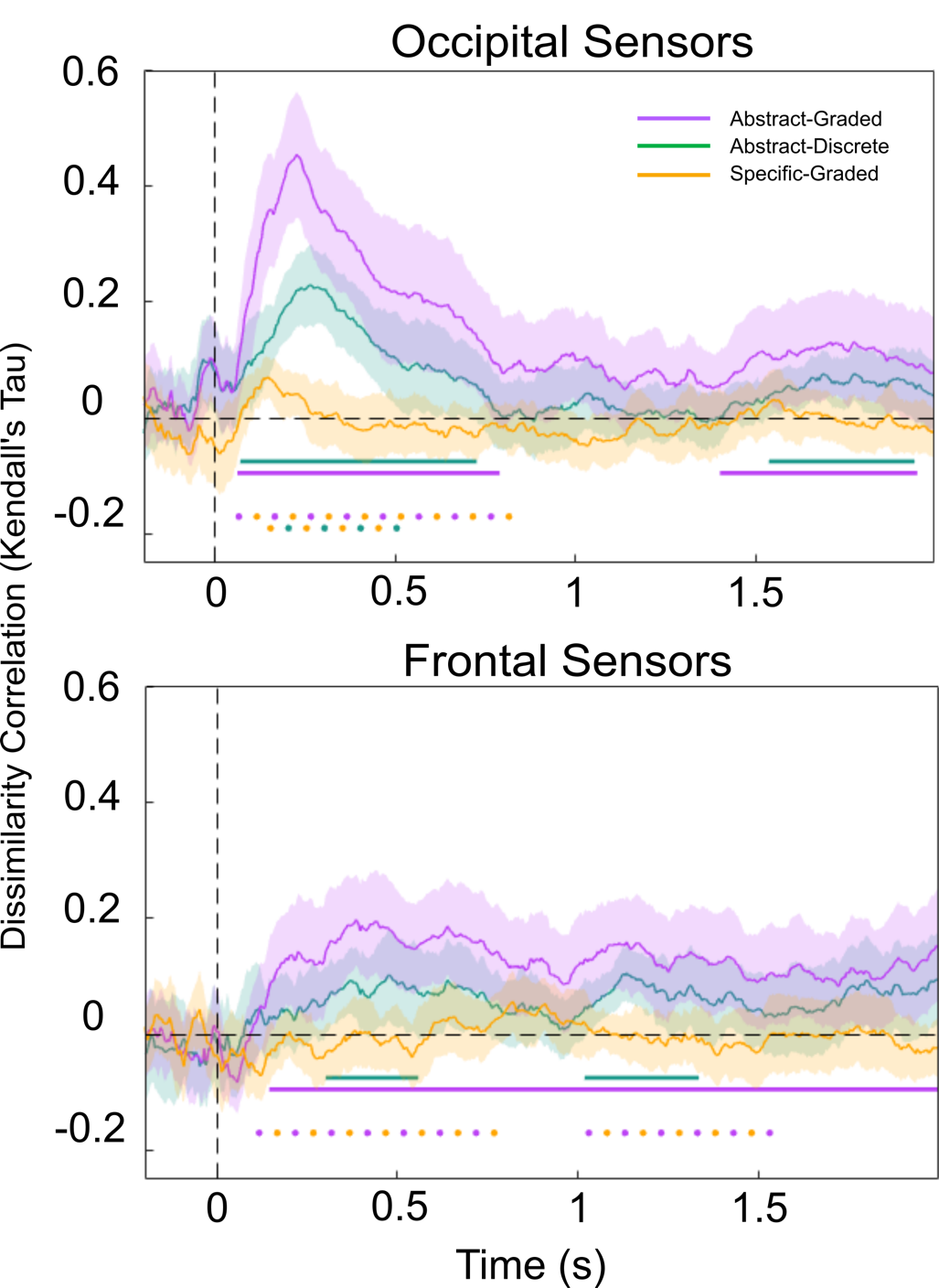


**Supplemental Figure 1. Awareness Ratings Show Similar Representational Structure Across Occipital and Frontal Sensors.** RSA analysis performed over occipital sensors (top) and frontal sensors (bottom) only. Purple, green, and gold lines represent similarity of the Abstract-Graded, Abstract-Discrete, and Specific-Graded models respectively with neural data. Solid horizontal lines represent time points significantly different from 0 for a specific RDM at p <.05, corrected for multiple comparisons. The Abstract-Graded model significantly predicted the neural data throughout the majority of the trial (purple line) across frontal sensors, and for a shorter duration when analysis was restricted occipital sensors only. The Abstract-Discrete model was only successful at predicting the neural data across two clusters of time-points post-stimulus when using occipital sensors, but was a significant predictor of neural data for larger portions of the epoch when using frontal sensors. The Specific-Graded model did not significantly predict the neural data at any time point in frontal or occipital sensors. Horizontal dots denote statistically significant paired comparisons between the different models at p <.05, corrected for multiple comparisons. Across frontal sensors, the Abstract-Graded model was a significantly better predictor of the neural data than the Specific-Graded model, and likewise across the occipital sensors, both abstract models significantly outperformed the Specific-Graded model. In this split-sensor analysis, the Abstract-Graded model did not significantly outperform the Abstract-Specific model, however there was a noticeable trend in the same direction as in the RSA performed across all sensors, where the difference in performance was significant (Figure 3B).


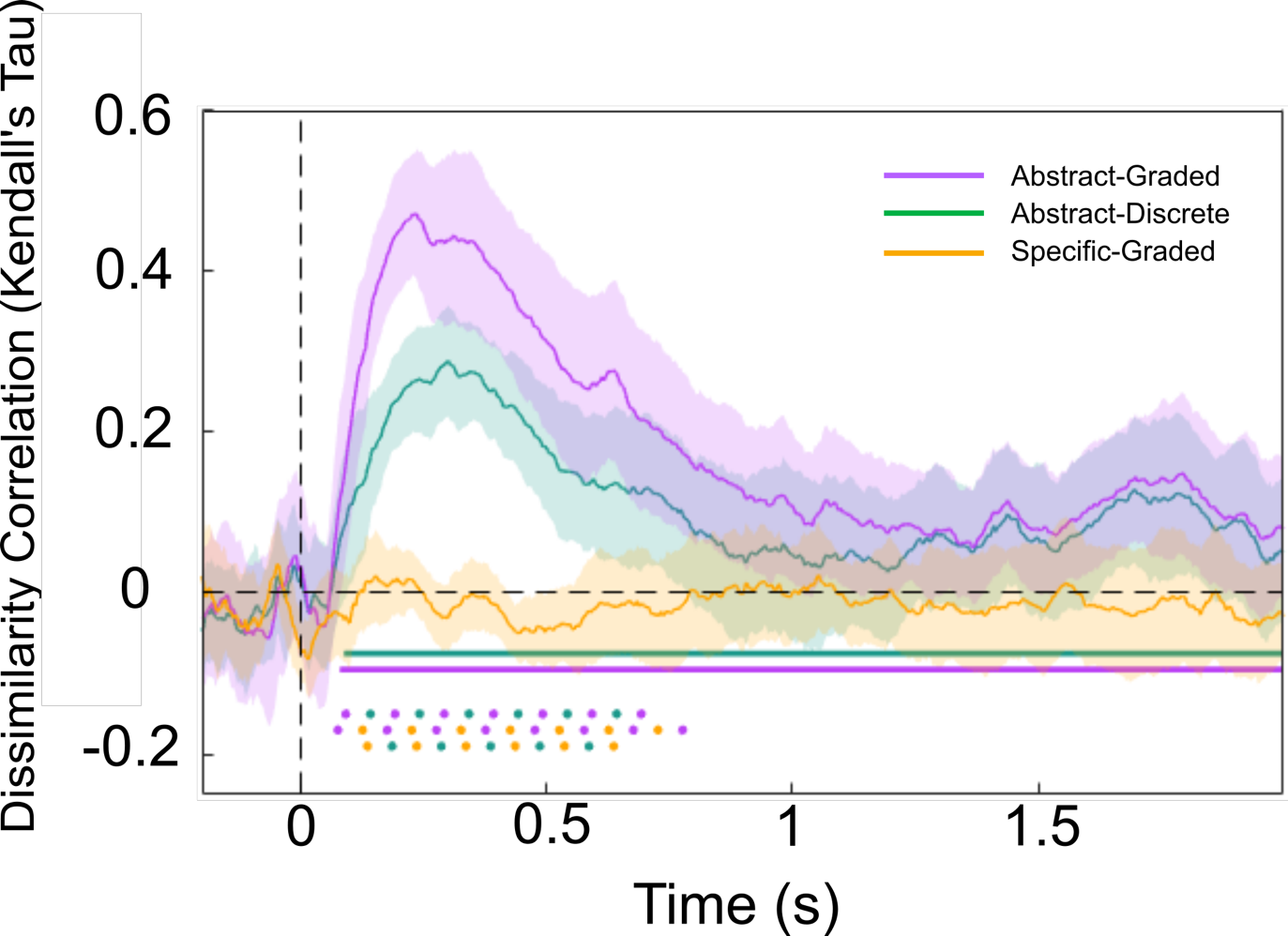


**Supplemental Figure 2. RSA on MEG data with stimulus contrast level regressed out.** When stimulus contrast level was regressed out of the MEG data, an RSA still produced comparable results to the original analysis. The Abstract-Graded model still predicted the neural data better than either alternative model. Solid horizontal lines represent time points significantly different from 0 for a specific RDM at p <.05, corrected for multiple comparisons. Horizontal dots denote statistically significant paired comparisons between the different models at p <.05, corrected for multiple comparisons.

**
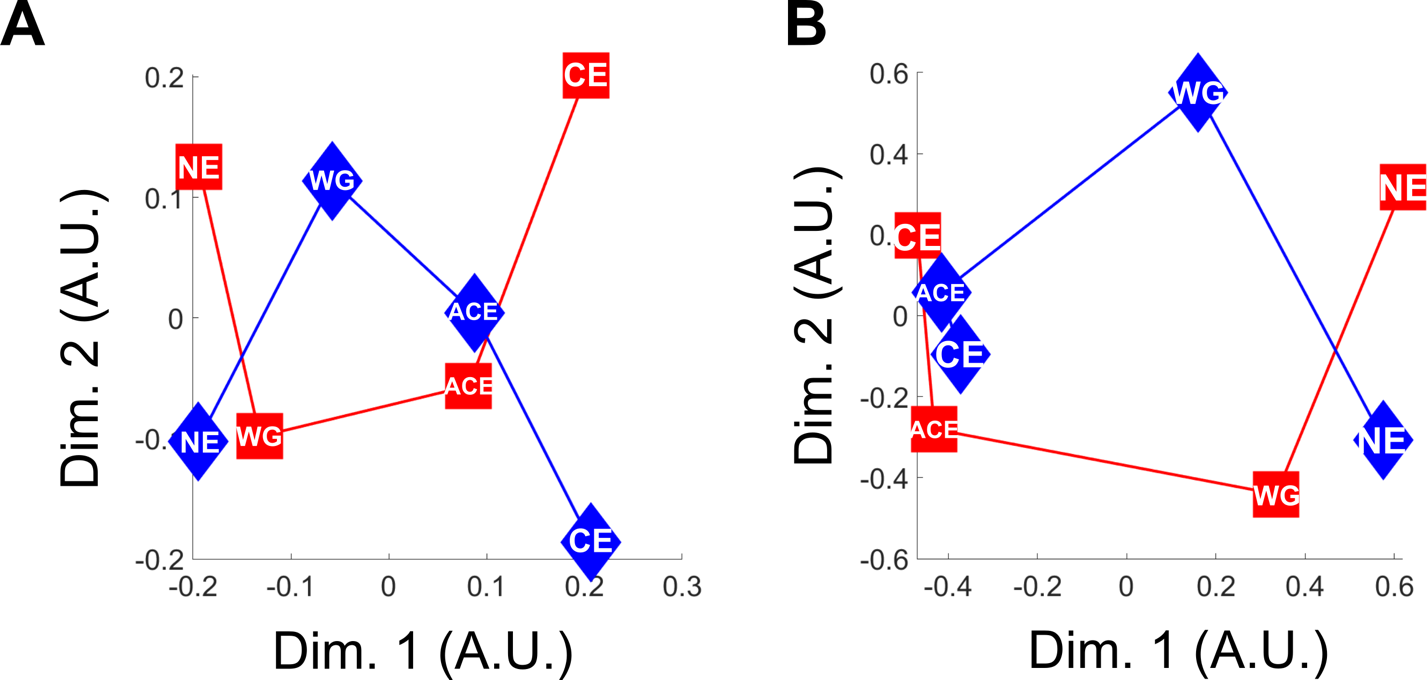
**

**Supplemental Figure 3.** Multidimensional scaling of neural activity covarying with awareness reports. A: Prior to stimulus contrast being regressed from the data, MEG activity covarying with awareness reports exhibit a linear trend from No Experience to Clear Experience along the first dimension, which tracks perceptual vividness. B: Following the removal of the linear component of stimulus contrast, the dimension which tracks perceptual vividness becomes compressed at the higher end, with ratings of “clear experience” (CE) and “almost clear experience” (ACE) becoming less distinct. Red squares illustrate ratings for squares, and blue diamonds illustrate ratings for diamonds. Data are averaged over the 100ms – 1000ms post-stimulus time window.


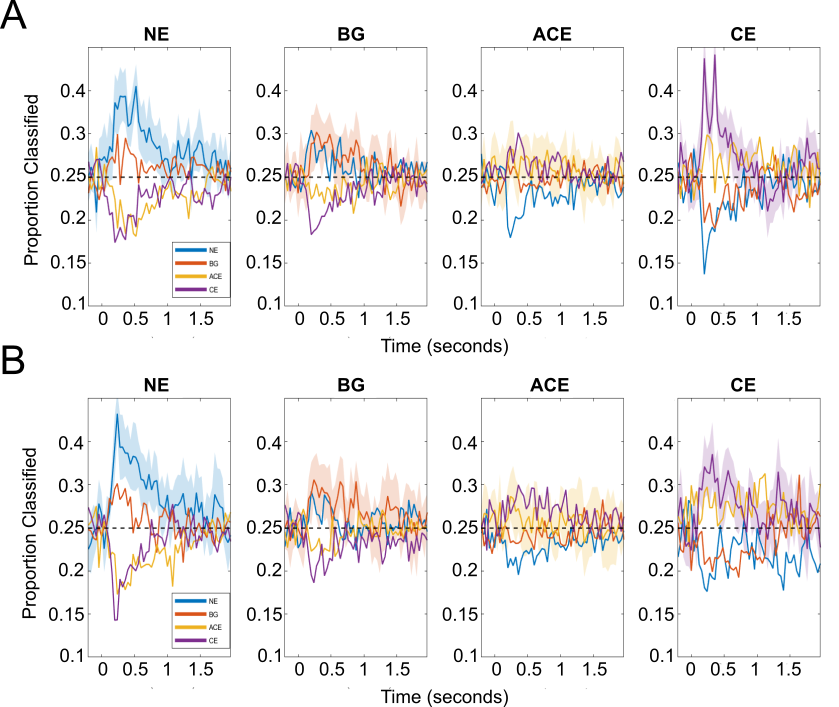


**Supplemental Figure 4: Cross-decoding shows the graded nature of the PAS scale.** For each cross-condition decoder (A: Train on Squares; B: Train on Diamonds), the four sub-plots illustrate the proportion of PAS ratings the classifiers decoded trials as. The subplots correspond to trials where the true PAS rating reported by subjects were (from left to right) ‘No Experience’, ‘Weak Glimpse’, ‘Almost Clear Experience’, and ‘Clear Experience’. Each coloured line represents the proportion of trials classified as each PAS rating across time. For example, in trials where participants reported No Experience, the majority of trials were classified correctly (blue line), with the classifier most often misclassifying these ‘No Experience’ trials as ‘Weak Glimpse’ ratings (orange line), and rarely misclassifying them as ‘Almost Clear Experience’ or ‘Clear Experience’ trials (gold and purple lines, respectively). Shaded areas represent 95% confidence intervals.


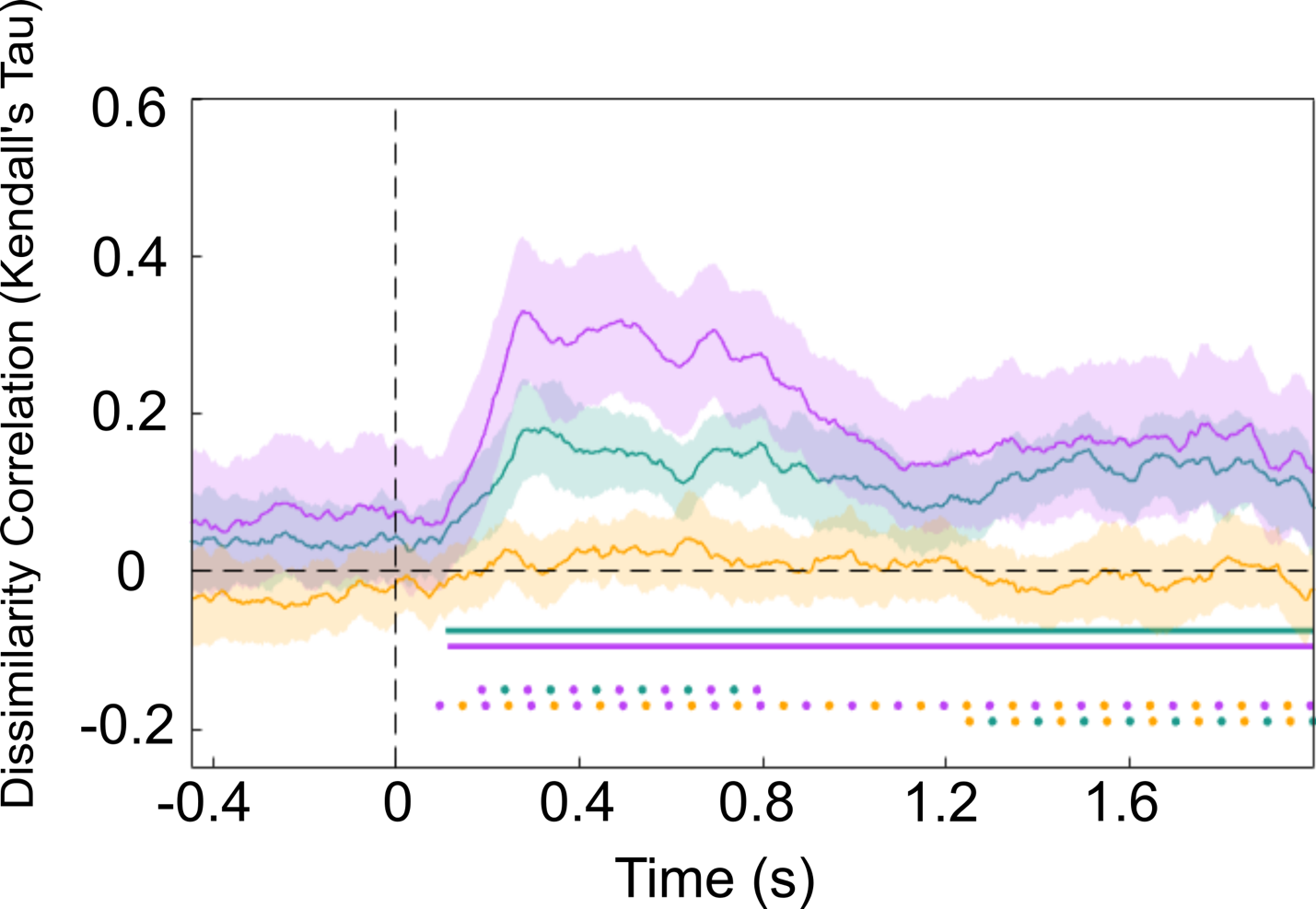


**Supplemental Figure 5. RSA analysis for MEG data without baseline correction.** None of our model RDMs significantly predicted pre-stimulus activity in non-baseline-corrected data. Instead, the predominant neural signature was stimulus triggered, as in the main analysis (Figure 3B), with the Abstract-Graded model being the best predictor of neural representations of phenomenal magnitude.

**
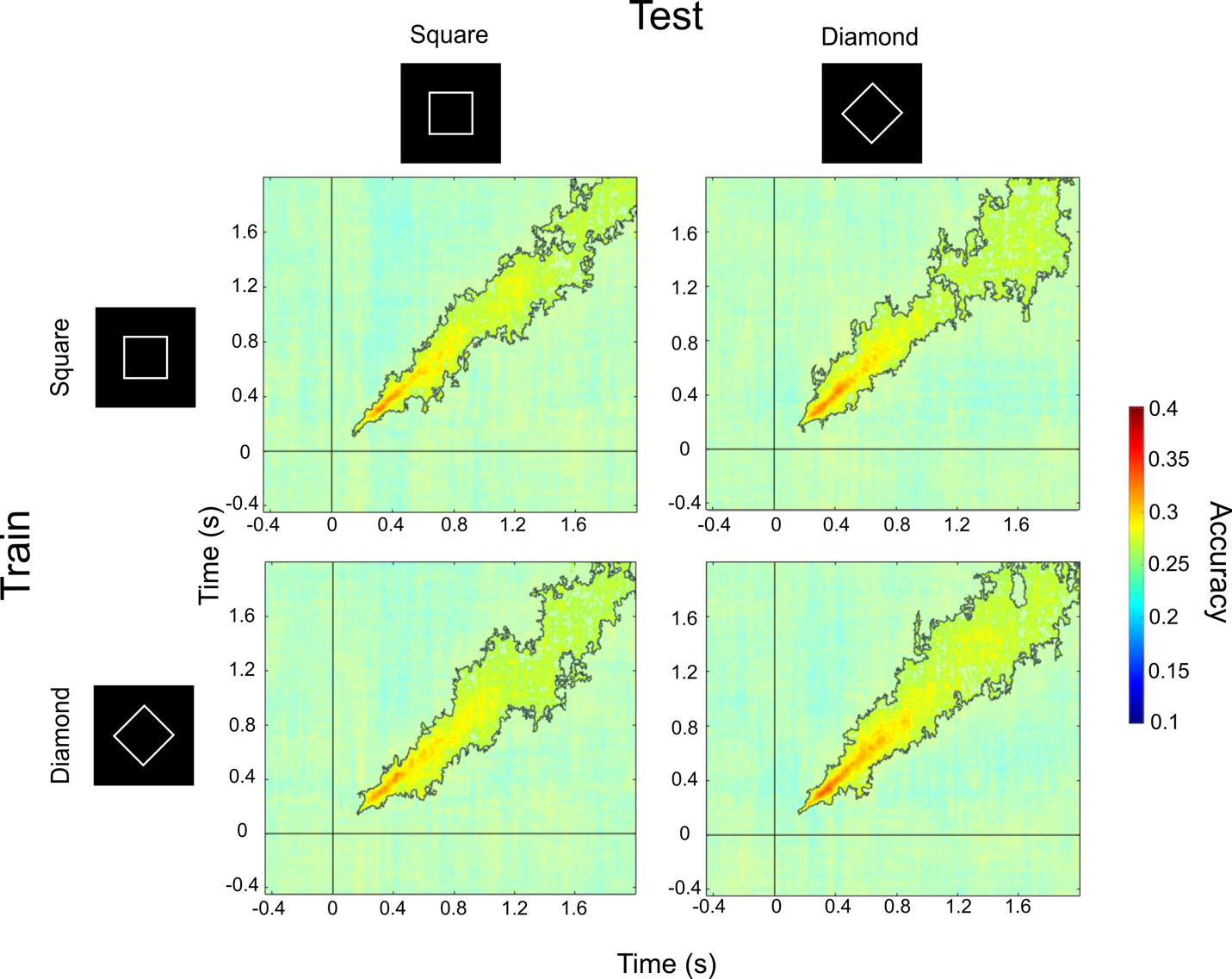
**

**Supplemental Figure 6. Temporal Generalisation matrices for MEG decoding analyses on data without baseline-correction.** For each row, statistical comparisons between the two columns showed no significant differences in decoding accuracy between within and cross-condition decoding. Pre-stimulus decoding of awareness ratings was not possible, even when data had not been baseline-corrected.

| **ROI** | **Cluster Size**  **(Number of Voxels)** | **MNI coordinates of Central Voxel**  **(X, Y, Z)** |
| --- | --- | --- |
| Visual | 52 | \| -21.9 \| -68 \| -15.7 \| \| --- \| --- \| --- \| |
| Visual | 51 | \| -19.7 \| -82 \| -20.1 \| \| --- \| --- \| --- \| |
| Visual | 33 | \| -35.9 \| -84.2 \| -20.1 \| \| --- \| --- \| --- \| |
| Visual | 19 | \| -41.8 \| -54 \| -23.8 \| \| --- \| --- \| --- \| |
| Visual | 5 | \| 26 \| -85.7 \| -20.1 \| \| --- \| --- \| --- \| |
| Frontal | 60 | \| -4.2 \| 30.1 \| 32.2 \| \| --- \| --- \| --- \| |
| Frontal | 51 | \| -4.2 \| 38.2 \| 18.2 \| \| --- \| --- \| --- \| |
| Frontal | 44 | \| 8.3 \| 26.7 \| 35.9 \| \| --- \| --- \| --- \| |
| Frontal | 10 | \| -21.9 \| 32.3 \| 38.1 \| \| --- \| --- \| --- \| |

**Table S1.** Clusters within both the visual and frontal regions of interest. Used for fMRI ROI decoding analyses. Clusters smaller than 5 voxels not shown.

| **Decoding Type** | **Atlas Label** | **Cluster Size (voxels)** | **MNI Coordinates of maximum accuracy** | **Maximum accuracy** |
| --- | --- | --- | --- | --- |
| Cross (train animate) | Postcentral Gyrus | 23,838 | -46, -36, 54 | 0.593 |
| Cross (train animate) | Temporal Fusiform Cortex | 474 | -31.5, -36.1, -25.8 | 0.567 |
| Cross (train animate) | Paracingulate Gyrus | 192 | -8, 44, -6 | 0.563 |
| Cross (train animate) | Frontal Orbital Cortex | 168 | -30, 34, -10 | 0.570 |
| Cross (train animate) | Cingulate Gyrus, posterior division | 109 | 4, -44, 10 | 0.563 |
| Cross (train animate) | Occipital Pole | 89 | -22, -96, 2 | 0.562 |
| Cross (train animate) | Frontal Pole | 75 | 32, 36, -10 | 0.561 |
| Cross (train animate) | Temporal Occipital Fusiform Cortex | 59 | 20, -52, -18 | 0.566 |
| Cross (train animate) | Lingual Gyrus | 56 | 10, -38, -6 | 0.555 |
| Cross (train animate) | Left Cerebral White Matter | 56 | -22, -38, 8 | 0.566 |
| Cross (train inanimate) | Supramarginal Gyrus, anterior division | 23,564 | -54, -36, 30 | 0.593 |
| Cross (train inanimate) | Subcallosal Cortex | 512 | 5.85, 10.6, -6.63 | 0.575 |
| Cross (train inanimate) | Left Caudate | 176 | -10, 2, 16 | 0.575 |
| Cross (train inanimate) | Middle Temporal Gyrus, posterior division | 166 | -52, -42, -2 | 0.585 |
| Cross (train inanimate) | Frontal Orbital Cortex | 152 | -32, 36, -12 | 0.570 |
| Cross (train inanimate) | Left Hippocampus | 106 | -20, -16, -14 | 0.567 |
| Cross (train inanimate) | Central Opercular Cortex | 100 | 38, 0, 18 | 0.571 |
| Cross (train inanimate) | Frontal Pole | 78 | 22, 42, -12 | 0.565 |
| Cross (train inanimate) | Insular Cortex | 71 | -38, -20, -4 | 0.564 |
| Cross (train inanimate) | Lateral Occipital Cortex, superior division | 62 | 32, -88, 14 | 0.555 |
| Within (animate) | Cingulate Gyrus, anterior division | 49,130 | -2, 36, 20 | 0.616 |
| Within (inanimate) | Postcentral Gyrus | 32,710 | 62, -8, 18 | 0.601 |
| Within (inanimate) | Right Caudate | 79 | 18, 10, 16 | 0.565 |

**Table S2. fMRI Searchlight Decoding Results.** Clusters with above chance decoding of perceptual visibility for both cross-condition and within-condition decoding. Clusters are significant at p < .05, corrected for multiple comparisons with an FDR of 0.01. Region names are found for the peak co-ordinate using the Harvard-Oxford Cortical and Subcortical Structural Atlas.

| **Decoding Type** | **Atlas Label** | **Cluster Size (voxels)** | **MNI Coordinates of maximum accuracy** | **Maximum accuracy** |
| --- | --- | --- | --- | --- |
| Content | Inferior Lateral Occipital Cortex | 2768 | -48, -70, -8 | 0.572 |
| Content | Cerebellum | 2150 | 0, -48, -10 | 0.558 |
| Content | Inferior Frontal Gyrus | 1837 | -52, 32, 6 | 0.556 |
| Content | Superior Lateral Occipital Cortex | 919 | -40, - 58, 54 | 0.557 |
| Content | Superior Lateral Occipital Cortex | 907 | -8, -64, 60 | 0.554 |
| Content | Angular Gyrus | 735 | 44, -52, 20 | 0.557 |
| Content | Superior Parietal Lobule | 626 | 20, -44, 62 | 0.548 |
| Content | Frontal Orbital Cortex | 375 | 38, 24, -2 | 0.557 |
| Content | Paracingulate Cyrus | 371 | -6, 26, 34 | 0.547 |
| Content | Inferior Frontal Gyrus | 356 | 46, 20, 20 | 0.563 |
| Content | Superior Frontal Gyrus | 209 | 16, 24, 58 | 0.546 |
| Content | Frontal Pole | 201 | -24, 42, 44 | 0.546 |
| Content | Superior Temporal Gyrus | 192 | -54, -2, -10 | 0.549 |
| Content | Postcentral Gyrus | 189 | -62, -22, 38 | 0.547 |
| Content | Posterior Cingulate Gyrus | 178 | -8, -26, 44 | 0.547 |
| Content | Left Cerebral White Matter | 133 | 6, -24, 16 | 0.544 |
| Content | Cerebellum | 98 | 34, -64, -40 | 0.547 |
| Content | Superior Lateral Occipital Cortex | 76 | 20, -66, 46 | 0.553 |
| Content | Anterior Cingulate Gyrus | 74 | 6, -4, 40 | 0.551 |
| Content | Parietal Operculum Cortex | 56 | 44, -32, 20 | 0.546 |
| Content | Precentral Gyrus | 52 | 44, -16, 58 | 0.542 |
| Mean Cross-Condition Visibility | Precentral Gyrus | 16,638 | -50, 8 32 | 0.584 |
| Mean Cross-Condition Visibility | Postcentral Gyrus | 2863 | -50, -30, 48 | 0.58 |
| Mean Cross-Condition Visibility | Right Accumbens | 278 | 10, 14, -6 | 0.561 |
| Mean Cross-Condition Visibility | Left Cerebral White Matter | 167 | -16, 0, 16 | 0.568 |
| Mean Cross-Condition Visibility | Frontal Medial Cortex | 166 | -4, 48, -10 | 0.555 |
| Mean Cross-Condition Visibility | Middle Temporal Gyrus | 150 | -52, -42, -2 | 0.556 |
| Mean Cross-Condition Visibility | Central Opercular Cortex | 138 | -36, -10, 18 | 0.563 |
| Mean Cross-Condition Visibility | Frontal Orbital Cortex | 133 | 32, 28, 2 | 0.556 |
| Mean Cross-Condition Visibility | Occipital Fusiform Gyrus | 116 | 14, -84, -22 | 0.557 |
| Mean Cross-Condition Visibility | Cerebellum | 103 | -28, -42, -34 | 0.559 |
| Mean Cross-Condition Visibility | Frontal Orbital Cortex | 95 | -36, 36, -10 | 0.563 |
| Mean Cross-Condition Visibility | Temporal Occipital Fusiform Cortex | 91 | -40, -62, -24 | 0.554 |
| Mean Cross-Condition Visibility | Inferior Lateral Occipital Cortex | 58 | 42, -80, 4 | 0.553 |
| Mean Cross-Condition Visibility | Frontal Pole | 58 | 22, 38, -12 | 0.557 |
| Mean Cross-Condition Visibility | Occipital Pole | 56 | 12, -90, -2 | 0.557 |
| Mean Cross-Condition Visibility | Superior Precentral Gyrus | 53 | 26, -12, 52 | 0.552 |
| Visibility and Content Overlap | Cerebellum | 1587 | -6, -82, -32 | N/A |
| Visibility and Content Overlap | Frontal Orbital Cortex | 1521 | -28, 16, -24 | N/A |
| Visibility and Content Overlap | Superior Lateral Occipital Cortex | 782 | -32, -74, 20 | N/A |
| Visibility and Content Overlap | Precuneous Cortex | 474 | -14, -58, 18 | N/A |
| Visibility and Content Overlap | Inferior Lateral Occipital Cortex | 412 | 46, -68, -10 | N/A |
| Visibility and Content Overlap | Supramarginal Gyrus | 383 | 64, -28, 34 | N/A |
| Visibility and Content Overlap | Frontal Pole | 339 | 40, 36, 12 | N/A |
| Visibility and Content Overlap | Cingulate Gyrus | 315 | 0, 36, 16 | N/A |
| Visibility and Content Overlap | Frontal Orbital Cortex | 213 | 36, 22, -14 | N/A |
| Visibility and Content Overlap | Middle Frontal Gyrus | 181 | -24, 36, 32 | N/A |
| Visibility and Content Overlap | Posterior Cingulate Gyrus | 156 | -4, -30, 36 | N/A |
| Visibility and Content Overlap | Middle Frontal Gyrus | 147 | 24, 28, 34 | N/A |
| Visibility and Content Overlap | Right Cerebral Cortex | 145 | 20, 2, -14 | N/A |
| Visibility and Content Overlap | Cerebellum | 115 | 26, -58, -26 | N/A |
| Visibility and Content Overlap | Cerebellum | 111 | -24, -36, -38 | N/A |
| Visibility and Content Overlap | Superior Parietal Lobule | 107 | 34, -50, 36 | N/A |
| Visibility and Content Overlap | Postcentral Gyrus | 101 | -60, -20, 24 | N/A |
| Visibility and Content Overlap | Postcentral Gyrus | 74 | -36, -22, 44 | N/A |
| Visibility and Content Overlap | Lingual Gyrus | 74 | -14, -56, -2 | N/A |
| Visibility and Content Overlap | Cerebellum | 63 | 20, -72, -32 | N/A |
| Visibility and Content Overlap | Cerebellum | 52 | 34, -56, -48 | N/A |

**Table S3. fMRI Searchlight Content Decoding in High Visibility Trials, Average Cross-Condition Visibility Decoding, and Overlapping Cluster Information.** *Decoding type = Content*: Clusters with above chance decoding of perceptual content (animate vs. inanimate) in high visibility trials. *Decoding type = Mean Cross-Condition Visibility*: Clusters with above chance decoding of perceptual visibility for cross-condition decoding when accuracy from both decoding directions (training on animate and training on inanimate) was averaged together. To aid the identification of individual clusters in this map, clustering was performed at an increased accuracy threshold of 0.54. *Decoding Type = Awareness and Content Overlap*: Clusters where decoding of content and cross-decoding of visibility were both successful (i.e. the intersection of the Content and Mean Cross-Condition Visibility clusters). Clusters are significant at p < .05, corrected for multiple comparisons with an FDR of 0.01. Region names are found for the peak co-ordinate using the Harvard-Oxford Cortical and Subcortical Structural Atlas and the MNI Structural Atlas.
