# Supplementary figures and images for: Identifying content-invariant neural signatures of perceptual vividness
Source: PNAS Nexus. 2024 Feb 14;3(2):pgae061. doi: 10.1093/pnasnexus/pgae061 (PMC10898512; doi:10.1093/pnasnexus/pgae061)

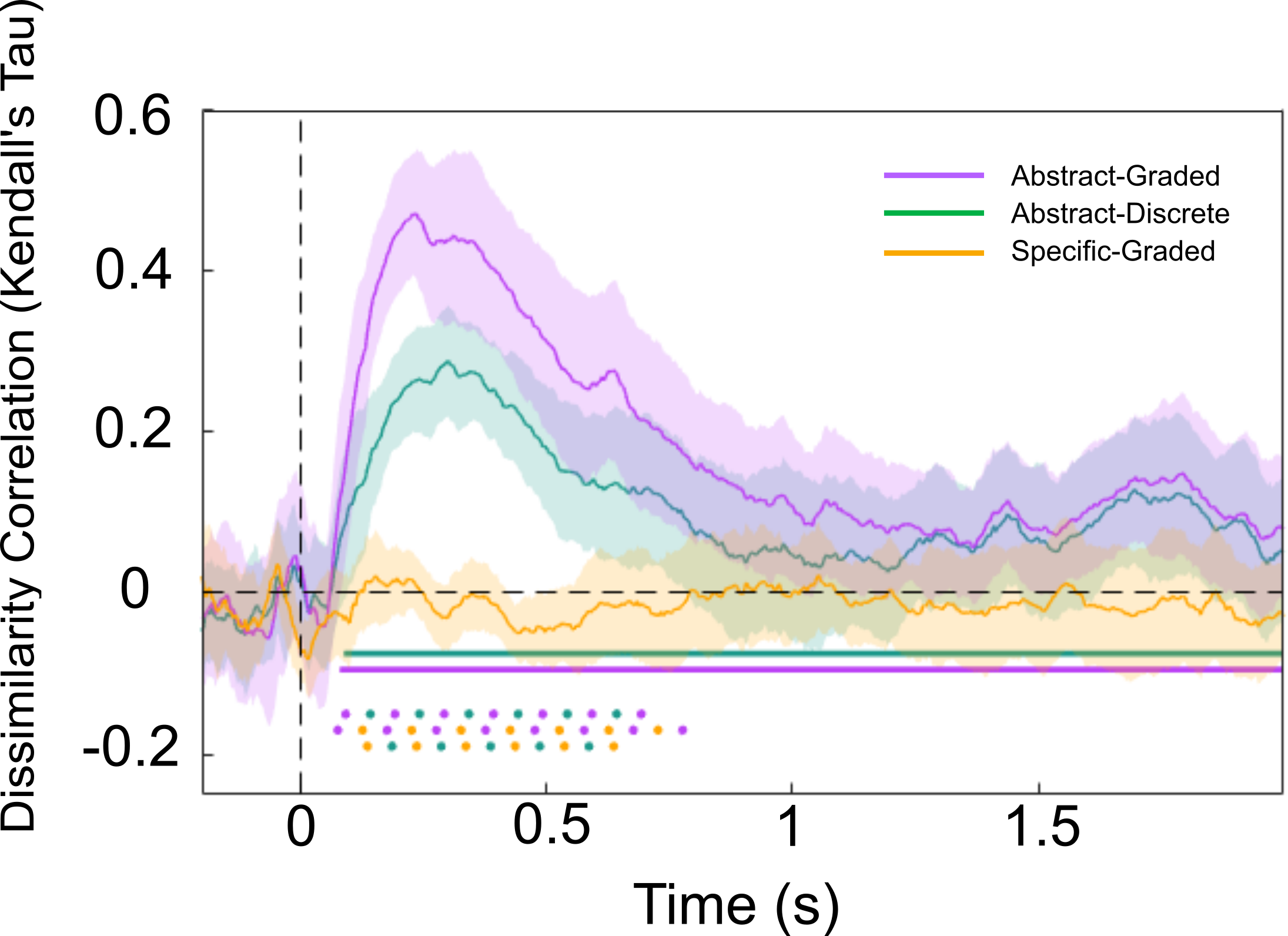

Supplement: pgae061_Supplementary_Data [file pgae061_supplementary_data.zip › PNASNEXUS-PNASNEXUS-2023-00872RR-s03.tif]

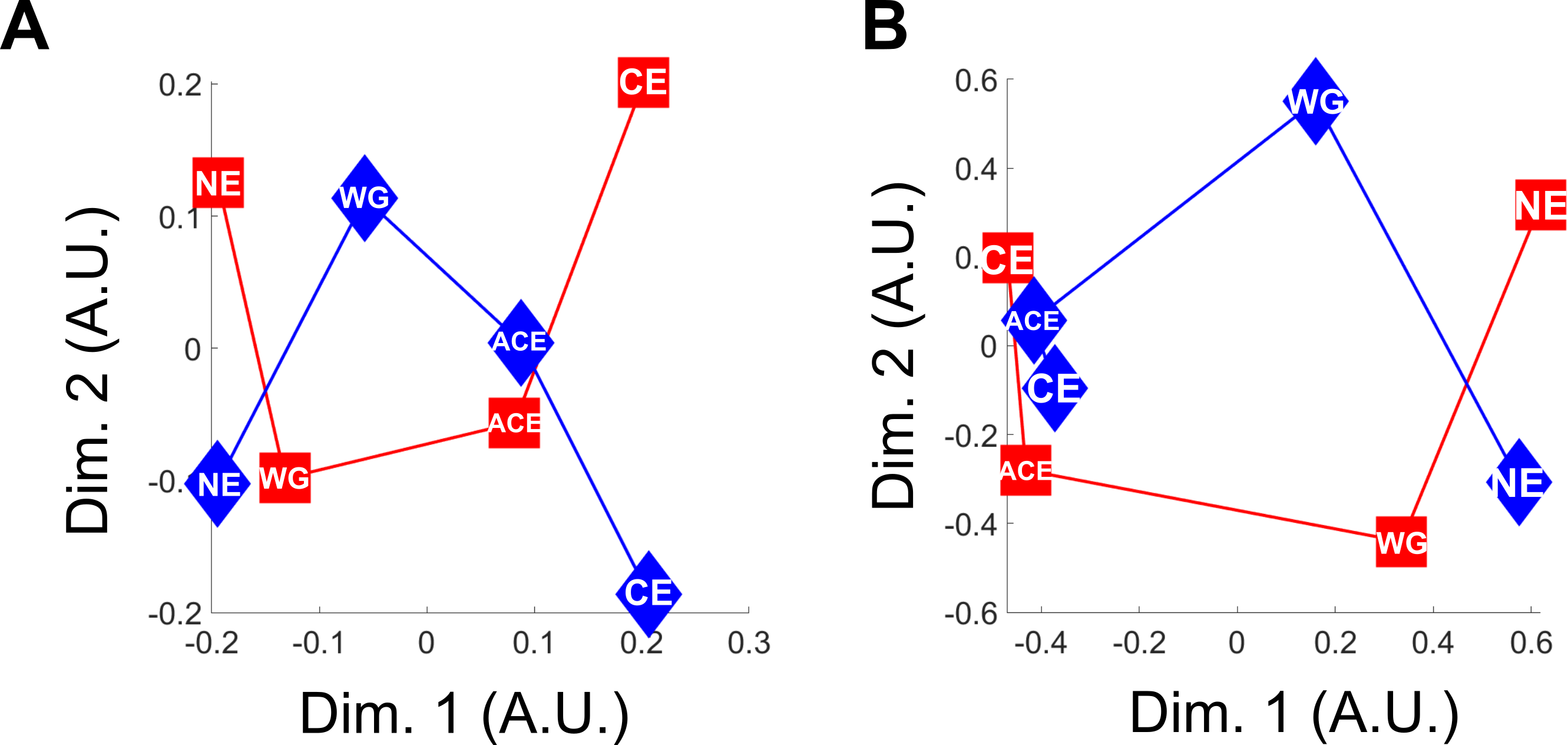

Supplement: pgae061_Supplementary_Data [file pgae061_supplementary_data.zip › PNASNEXUS-PNASNEXUS-2023-00872RR-s04.tif]

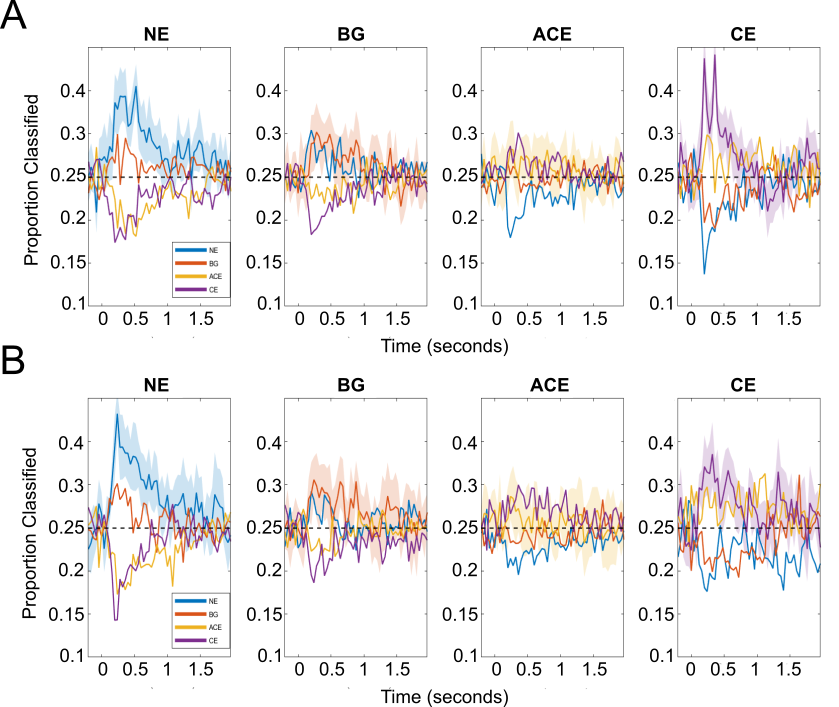

Supplement: pgae061_Supplementary_Data [file pgae061_supplementary_data.zip › PNASNEXUS-PNASNEXUS-2023-00872RR-s05.tif]

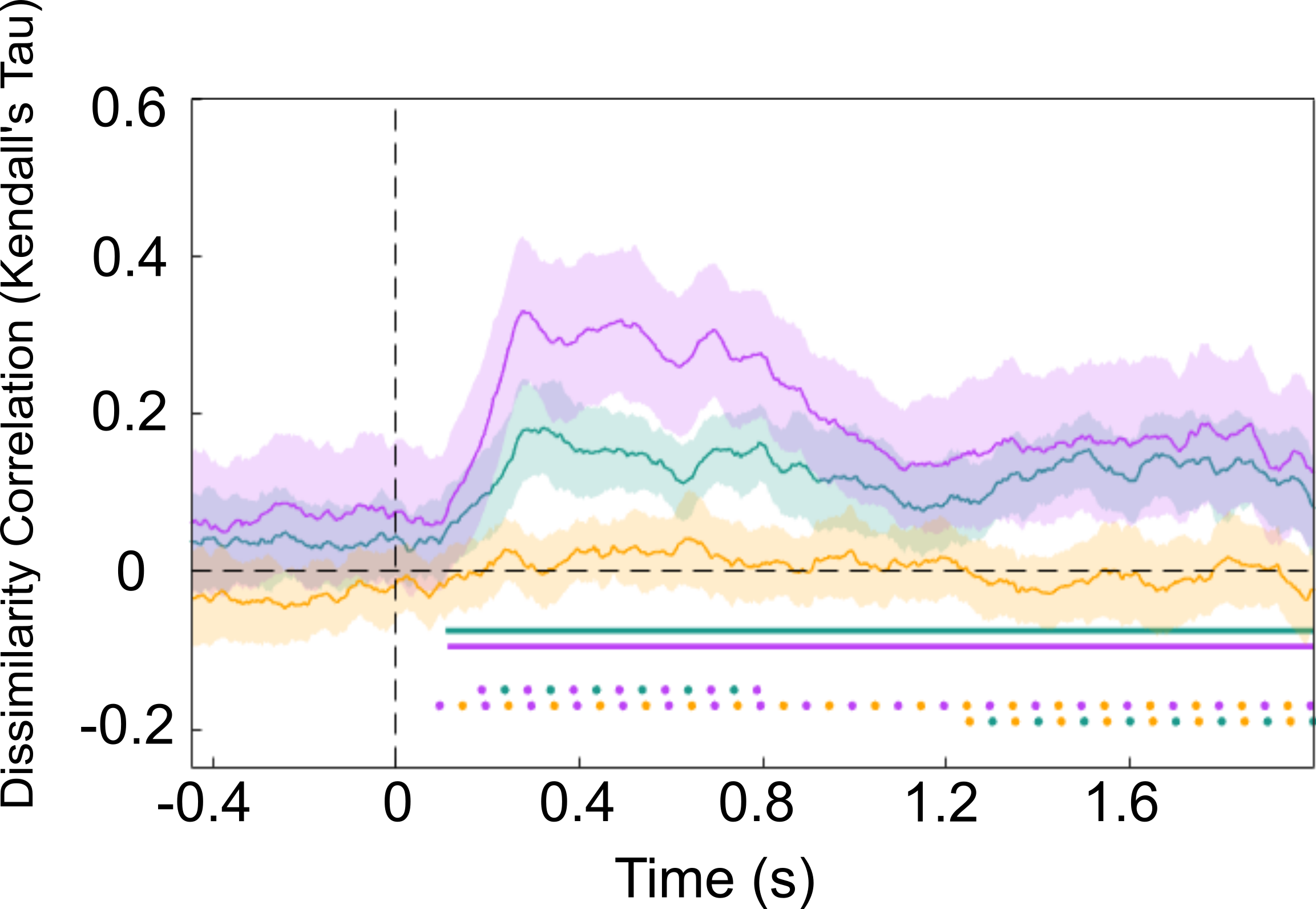

Supplement: pgae061_Supplementary_Data [file pgae061_supplementary_data.zip › PNASNEXUS-PNASNEXUS-2023-00872RR-s06.tif]

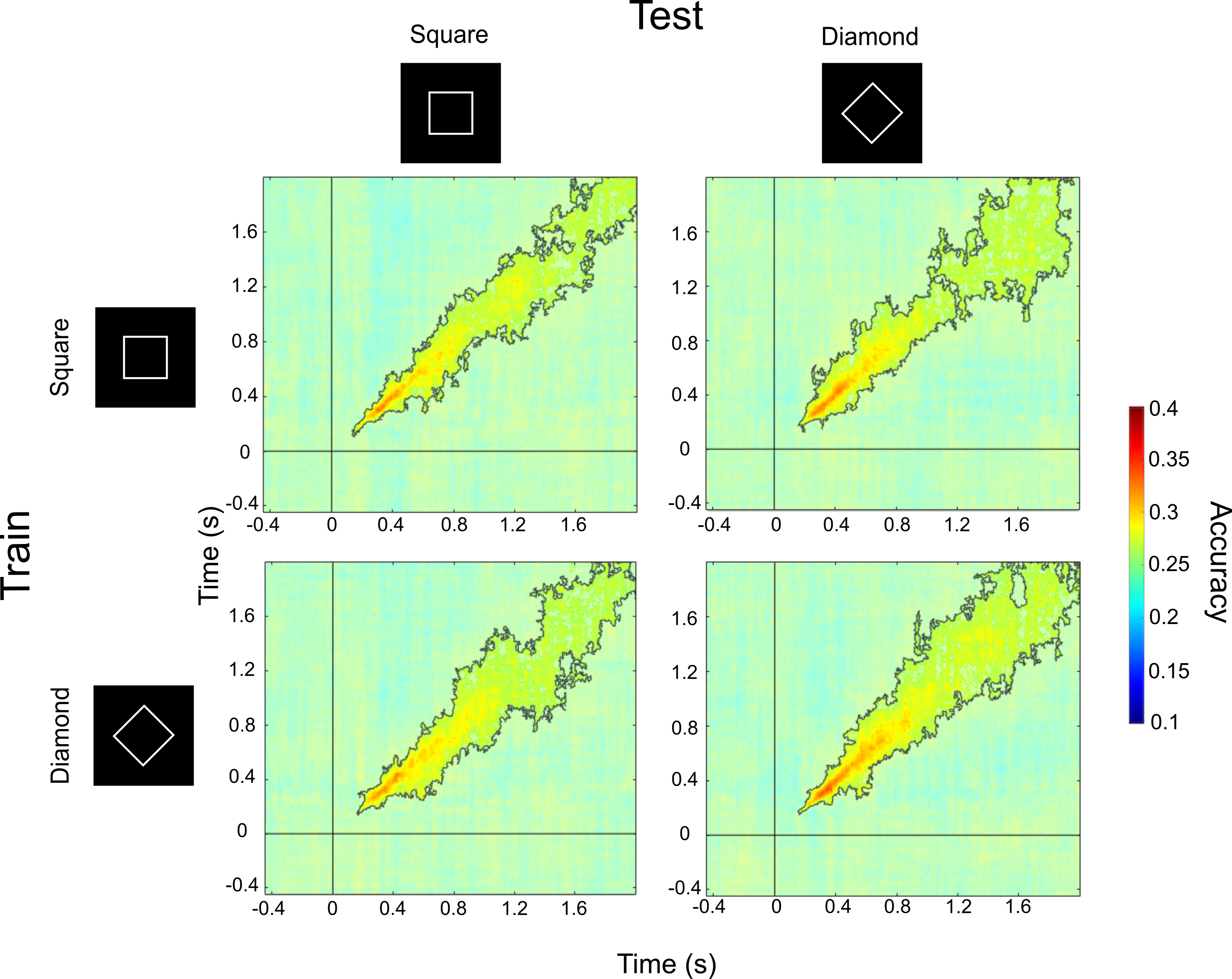

Supplement: pgae061_Supplementary_Data [file pgae061_supplementary_data.zip › PNASNEXUS-PNASNEXUS-2023-00872RR-s07.tif]

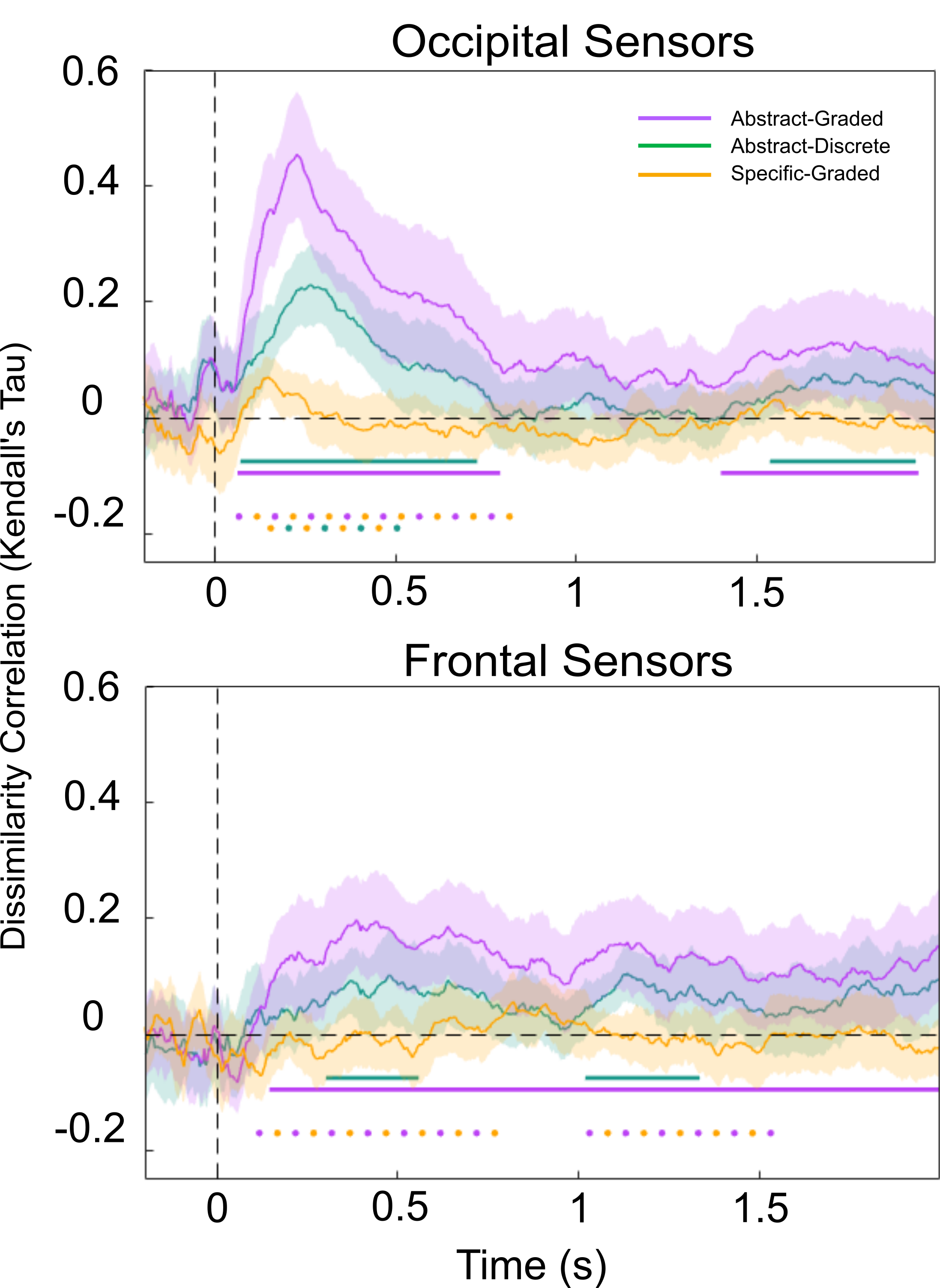

Supplement: pgae061_Supplementary_Data [file pgae061_supplementary_data.zip › PNASNEXUS-PNASNEXUS-2023-00872RR-s02.tif]
